# Counseling Supporting HIV Self-Testing and Linkage to Care Among Men Who Have Sex With Men: Systematic Review and Meta-Analysis

**DOI:** 10.2196/45647

**Published:** 2024-01-24

**Authors:** Siyu Chen, Yuan Fang, Paul Shing-fong Chan, Joseph Kawuki, Phoenix Mo, Zixin Wang

**Affiliations:** 1 Centre for Health Behaviours Research Jockey Club School of Public Health and Primary Care The Chinese University of Hong Kong Hong Kong China (Hong Kong); 2 Department of Health and Physical Education The Education University of Hong Kong Hong Kong China (Hong Kong)

**Keywords:** HIV self-testing, counseling, linkage to care, men who have sex with men, meta-analysis, mobile phone

## Abstract

**Background:**

Counseling supporting HIV self-testing (HIVST) is helpful in facilitating linkage to care and promoting behavior changes among men who have sex with men (MSM). Different levels of counseling support for MSM HIVST users may lead to variance in the linkage to care.

**Objective:**

This study aims to synthesize evidence on counseling supporting MSM HIVST users and to conduct a meta-analysis to quantify the proportion of MSM HIVST users who were linked to care.

**Methods:**

A systematic search was conducted using predefined eligibility criteria and relevant keywords to retrieve studies from the MEDLINE, Global Health, Web of Science, Embase, APA PsycINFO, and Scopus databases. This search encompassed papers and preprints published between July 3, 2012, and June 30, 2022. Studies were eligible if they reported counseling supporting HIVST or quantitative outcomes for linkage to care among MSM and were published in English. The screening process and data extraction followed the PRISMA (Preferred Reporting Items for Systematic Reviews and Meta-Analyses) guidelines. The quality of the included studies was assessed by the National Institutes of Health quality assessment tool. Data were extracted using random effects models to combine the proportion of HIVST users who were linked to care. Subgroup analyses and metaregression were conducted to assess whether linkage to care varied according to study characteristics. All analyses were performed with R (version 4.2.1; R Foundation for Statistical Computing) using the *metafor* package.

**Results:**

A total of 55 studies published between 2014 and 2021, including 43 observational studies and 12 randomized controlled trials, were identified. Among these studies, 50 (91%) provided active counseling support and 5 (9%) provided passive counseling support. In studies providing active counseling support, most MSM HIVST users were linked to various forms of care, including reporting test results (97.2%, 95% CI 74.3%-99.8%), laboratory confirmation (92.6%, 95% CI 86.1%-96.2%), antiretroviral therapy initiation (90.8%, 95% CI 86.7%-93.7%), and referral to physicians (96.3%, 95% CI 85%-99.2%). In studies providing passive counseling support, fewer MSM HIVST users were linked to laboratory confirmation (78.7%, 95% CI 17.8%-98.4%), antiretroviral therapy initiation (79.1%, 95% CI 48.8%-93.7%), and referral to physicians (79.1%, 95% CI 0%-100%). Multivariate metaregression indicated that a higher number of essential counseling components, a smaller sample size (<300), and the use of mobile health technology to deliver counseling support were associated with better linkage to care. The quality of the studies varied from fair to good with a low to high risk of bias.

**Conclusions:**

Proactively providing counseling support for all users, involving a higher number of essential components in the counseling support, and using mobile health technology could increase the linkage to care among MSM HIVST users.

**Trial Registration:**

PROSPERO CRD42022346247; https://www.crd.york.ac.uk/prospero/display_record.php?RecordID=346247

## Introduction

### Background

Globally, the estimated median HIV prevalence among men who have sex with men (MSM) ranges from 5% in Southeast Asia to 12.6% in Eastern and Southern Africa [1]. The risk of acquiring HIV is 26 times higher among MSM compared with the general population worldwide [1]. In 2022, the proportion of new HIV cases attributed to MSM was 44% in Asia and the Pacific [2], 38.7% in Europe, and 70% in the United States [3-5].

HIV testing is one of the key strategies for controlling the spread of HIV [6,7]. Both the Joint United Nations Programme on HIV/AIDS (UNAIDS) and the Centers for Disease Control and Prevention recommend MSM to undergo HIV testing every 3 to 6 months [6,7]. The UNAIDS established its 95-95-95 targets in 2014 [6]. The aim was to diagnose 95% of all individuals testing positive for HIV, provide antiretroviral therapy (ART) to 95% of those diagnosed, and achieve viral suppression for those treated by 2030 [6]. High coverage of HIV testing was the first step in achieving the 95-95-95 targets. However, the overall HIV testing coverage among MSM was 86.2% in Africa and 89% in North America [8]. There is a need for further improvement. HIV self-testing (HIVST) could serve as an alternative strategy for enhancing HIV testing coverage. Systematic reviews have demonstrated that HIVST can overcome barriers faced by MSM when accessing HIV testing services, such as perceived stigma from providers and inconvenience [9-11]. Previous meta-analyses consistently showed that HIVST has doubled the frequency of HIV testing compared with facility-based testing [12,13]. As a result, the World Health Organization (WHO) recommends offering HIVST as an additional approach in addition to the existing HIV testing services [14].

On the basis of the presence of counseling support, HIVST can be categorized into assisted and unassisted HIVST. Several studies investigated the linkage to care in assisted and unassisted HIVST. Individuals who received positive results through unassisted HIVST faced more difficulties in accessing care than those who were identified by facility-based HIV testing and counseling [15]. According to a systematic review, <25% of unassisted HIVST users were able to complete the test without any errors, and many of them had difficulties interpreting the HIVST results [16]. A meta-analysis showed that the absence of assistance would lead to a 17% decrease in the linkage to care rate among HIVST users [12]. Across countries, studies have consistently shown that implementing assisted HIVST could increase linkage to care among different populations [17-19]. A very high linkage to care (99%-100%) was observed among users of assisted HIVST in the United States and Zimbabwe [19]. Therefore, the WHO recommends that counseling support be provided to HIVST users [20].

As recommended by the WHO, 8 essential components should be included in the pretest and posttest counseling of a standard-of-care client-initiated HIV testing and counseling. The pretest counseling should include (1) assessing the risks and window periods, (2) informing clients of the benefit of taking the test and the implications of both negative and positive results, (3) assuring the clients’ right to refuse to take the test, (4) encouraging the clients to anticipate the possibility of beneficial disclosure of serostatus status, and (5) providing preventive information and materials [21]. Essential components of posttest counseling include (1) interpreting testing results; (2) offering psychological support to individuals testing positive for HIV, facilitating beneficial disclosure of their positive serostatus, and referring them for further care and support services; and (3) providing HIV-negative individuals with preventive information and materials [21]. However, the level of counseling support varied across previous HIVST programs. Some programs proactively provided pretest or posttest counseling support to all HIVST users unless they refused [22]. This mode of counseling support was categorized as active counseling [23-25]. Providing active counseling increased ART adherence among people living with HIV who had an unsuppressed viral load [26]. Other programs did not provide active counseling to users. Users could report their results via a web-based platform and obtain optional posttest counseling [27]. This mode of counseling support was categorized as passive counseling [24,25]. In addition, the number of essential components involved in counseling support varied significantly. Some studies only involved a single essential component (eg, providing additional HIV care for HIVST users who received reactive results), whereas others provided more comprehensive support (eg, assessing the risks and window periods, delivering preventive information and materials, and providing additional HIV care for HIVST users who received reactive results) [26,27].

### Objectives

Existing systematic reviews and meta-analyses have investigated the digital support [28], effectiveness [29], and acceptability of HIVST [30]. However, few studies have summarized the levels of counseling support for HIVST among MSM. It is also unclear whether different levels of counseling support would result in differences in the linkage to care among MSM HIVST users. To address this knowledge gap, we systematically reviewed global evidence on counseling support for MSM HIVST users. We also summarized the linkage to care under different modes of counseling support, including (1) the proportion of users who reported HIVST results; (2) the proportion of users with reactive results who were linked to laboratory confirmation, ART initiation, and physicians; and (3) the proportion of users with negative results who were given information related to sexual risk behavior reduction, related to pre-exposure prophylaxis (PrEP), and linked to PrEP initiation.

## Methods

This systematic review and meta-analysis was registered with PROSPERO (CRD42022346247) and conducted according to the PRISMA (Preferred Reporting Items for Systematic Reviews and Meta-Analyses) guidelines (Multimedia Appendix 1) [31].

### Search Strategy

We searched the MEDLINE, Global Health, Web of Science, Embase, APA PsycINFO, and Scopus databases for studies (including both published papers and preprints) between July 3, 2012 (the date when HIVST was approved by the Federal Drug Administration), and June 30, 2022, in any country or setting [32]. Keywords were selected based on the PICOS (participants, intervention, comparison, outcome, and study) criteria to address the research question (where P=MSM, I=HIVST with counseling, C=none, O=linkage to care, and S=randomized controlled trial [RCT] or observational studies). The Boolean operator was used in the search strategy, with “OR” and/or “AND” used to link search terms, whereas the asterisk “*” was used as a wildcard symbol appended at the end of the terms to search for variations of those terms. Full search strategies are available in Multimedia Appendix 2.

Additional studies were identified through the UNAIDS and WHO websites. We also reviewed databases listing ongoing RCTs, such as ClinicalTrials.gov, the WHO International Clinical Trials Registry Platform, and the Pan African Clinical Trials Registry, as well as reference lists of published reviews, meta-analyses, and articles.

### Inclusion and Exclusion Criteria

Inclusion and exclusion criteria are presented in Table 1. The exposure categories and outcomes of the study are presented in Textbox 1.

**Table 1:** Summary of the inclusion and exclusion criteria.

Parameter	Inclusion criteria	Exclusion criteria
Article or study type	Population-based original research studies Quantitative studies Multicountry studies Gray literature and preprints	Reviews, narratives, commentaries, and editorials Qualitative studies Dissertations, government reports, newspaper articles, textbooks, book chapters, and protocols Laboratory studies, model and framework studies, and validation studies
Language	English language	All other non-English languages
Publication period	July 3, 2012, to June 30, 2022	All periods outside July 3, 2012, to June 30, 2022
Study setting	All countries	None

**Exposure categories and outcomes included in the review of linkage to care following HIV self-testing (HIVST), along with counseling.**

**Exposure categories**

1. Studies that included HIVST along with active counseling support (eg, studies proactively provided pretest or posttest counseling to all HIVST users unless refused)
2. Studies that included HIVST along with passive counseling support (eg, studies provided certain level of counseling support to HIVST users upon request)

**Outcomes**

1. Reporting test results (defined as the proportion of men who have sex with men [MSM] who reported test results)
2. Laboratory confirmation (defined as the proportion of receiving confirmatory test among MSM with reactive HIVST results)
3. Antiretroviral therapy (ART) initiation (defined as the proportion of ART initiation among MSM HIVST users who are confirmed to be HIV positive)
4. Referred to physicians (defined as the proportion of seeking physicians among MSM HIVST users who are confirmed to be HIV positive)
5. Prevention strategies (defined as the proportion of MSM with negative HIVST results who received information related to sexual risk behaviors reduction, pre-exposure prophylaxis [PrEP])
6. PrEP initiation (defined as the proportion of starting PrEP among MSM with negative HIVST results who had the risk of HIV infection)

### Data Extraction

Critical information from this study was extracted using a data extraction form, as outlined in Table 2. The study outcome was the proportion of MSM HIVST users who were linked to care. Two independent reviewers (SC and YF) assessed the eligibility, evaluated the quality, and extracted information from the included publications. Any disagreements during the data extraction and quality assessment process were resolved by a senior reviewer (ZW).

**Table 2:** Characteristics, active counseling, and passive counseling support of included studies.

Study	Study setting or country	Study design	Sample size	Age (y)	Counseling delivering modes	Active counseling support	Passive counseling support
Marlin et al [33], 2014	United States	Cross-sectional study	641	90% of the participants were aged between 18 and 35 years	Technology or mobile health	They were provided linkage-to-care consultation via telephone survey	N/A^a^
Tao et al [34], 2014	China	Cross-sectional study	220	Median 28 (IQR 22-29)	Technology or mobile health	They were provided pretest and posttest counseling via a telephone hotline or QQ Group and instructions by a web-based video posted to the website.	N/A
Sabharwal et al [35], 2015	United States	Cross-sectional study	53	Mean 32	Peer or community	They were provided partner services and linkage to care to all persons newly diagnosed with HIV by New York City health department.	N/A
Huang et al [36], 2016	United States	Cross-sectional study	334	65% of the participants were aged between 18 and 30 (range 18 to >41)	Technology or mobile health	They were provided test result instructions, linkage-to-care activities via email or SMS text message, and 69% of the participants were satisfied the service.	N/A
Rosengren et al [37], 2016	United States	Cross-sectional study	125	93% of the participants were aged between 18 and 40 (range 18 to >41)	Technology or mobile health	They were provided reminder emails to complete a web-based survey to report testing results and posttest counseling, and 77% of the participants were satisfied the service.	N/A
Volk et al [38], 2016	Brazil	Cohort study	103	51% of the participants were aged between 18 and 25 (range 18 to >25)	Technology or mobile health	They were provided written instructions that included pictures, pretest and posttest counseling materials, a list of local HIV/AIDS resources, and condoms by mobile phones or email access, and 98% of the participants were satisfied the service.	N/A
Jamil et al [39], 2017	Australia	RCT^b^	178	Mean 35.8 (SD 11.1)	Technology or mobile health	They were provided expedited confirmatory testing, clinical review, and supportive counseling to any participant with a reactive self-test result at the study clinics, and 90% of the participants were satisfied the service.	N/A
Qin et al [40], 2017	China	Cross-sectional study	341	Mean 24.4 (SD 6.3)	Peer or community	They were provided confirmation of self-test results (at a CDC^c^ or hospital), posttest counseling, and potential harms (coercion, feelings of suicidality, and violence), and 47% of participants were satisfied the service.	N/A
Zhong et al [41], 2017	China	Cross-sectional study	198	54.2% of the participants were aged between 25 and 34 (range 18 to >35)	Technology or mobile health	They were provided the results interpretation, counseling services, confirmation testing, and linkage to care.	N/A
Choko et al [42], 2018	Uganda	Cohort study	95	Median 41 (IQR 23-62)	Peer or community	They were provided results interpretation and the opportunity to undergo confirmatory HIV testing, and 99% of the participants were satisfied the service.	N/A
Green et al [43], 2018	Vietnam	Cross-sectional study	803	Most of the participants were aged ≤30 years	Peer or community	They were provided confirmatory testing at the closest district health center, and those that were HIV diagnosed were helped with treatment enrollment by peer and staff.	N/A
Katz et al [44], 2018	United States	RCT	230	Median 35.5 (IQR 27-45.5)	Peer or community	They were provided instructions, pretest and posttest counseling materials, a list of local HIV-related resources, and condoms.	N/A
Lippman et al [45], 2018	South Africa	Cohort study	127	65% of the participants were aged between 18 and 24 years	Technology or mobile health	They were provided with logs to document the use of the tests, a list of local psychosocial and medical resources and referrals should the participant test HIV positive—including a 24-hour study phone number—and safer sex supplies (ie, condoms and lubricant), and 97% of participants were satisfied the service.	N/A
Pant Pai et al [46], 2018	Canada	Cross-sectional study	451	Mean 34 (range 18-73)	Technology or mobile health	They were provided instructions on pretest counseling, staging, conducting results, and storing their results on from *HIVSmart* app, and 99% of participants were satisfied the service.	N/A
Tun et al [47], 2018	Nigeria, West Africa	Cohort study	319	Median 25	Peer or community	They were provided information on HIVST^d^ kit use, counseling, and referrals for HIV care and treatment and other support services by a certified HIV testing counselor provide.	N/A
Wray et al [48], 2018	United States	RCT	65	Mean 34.1 (SD 13.9), range 18-72	Technology or mobile health	They were provided pretest counseling by opening their test kits within 24 hours to answer any questions and offer referrals to other sexual health services over the phones and posttest counseling referral within 24 hours after 100% of detected tests.	N/A
Wang et al [49], 2018	China	RCT	430	63.1% of the participants were aged between 18 and 30 years	Technology or mobile health	They were provided standard-of-care pretest and posttest counseling by the administrators, and 81% of participants were satisfied the service.	N/A
Jin et al [50], 2019	China	Cross-sectional study	879	Median 28 (IQR 24-34)	Technology or mobile health	N/A	They were provided with a peer navigator to accompany them to receive confirmatory testing, as well as initial visits for treatment and care following formal diagnosis if they were interested in linkage services.
De Boni et al [51], 2019	Brazil	Cross-sectional study	2526	Median 25 (IQR 22-31)	Technology or mobile health	They were provided free anonymous HIVST and to enhance linkage to HIV care for those with a confirmed HIV positive status by an internet-based HIVST [electronic testing (e-testing)] approach.	N/A
Gashobotse [52], 2019	Burundi, East Africa	Cross-sectional study	231	18-50	Peer or community	They were provided support, and confirmatory testing by peer educators and health care workers.	N/A
Hidayat et al [27], 2019	Indonesia	Cross-sectional study	317	Mean 29.9	Peer or community	N/A	They were provided interpreting the results and confirmatory test only determining how many lines appear on the result display.
Nguyen et al [53], 2019	Vietnam	Cross-sectional study	2185	44.4% of the participants were aged between 16 and 25 years	Peer or community	They were provided results interpretation and observed by peer educators.	N/A
Vera et al [54], 2019	United Kingdom	Cross-sectional study	232	37% of the participants were aged between 45 and 64 (range 25-65) years	Technology or mobile health	They were provided a detailed information about linkage to care (contact for the nearest sexual health clinic) and support in case of a reactive result (helpline details), and 94% of the participants were satisfied the service.	N/A
Wesolowski et al [55], 2019	United States	Cohort study	80	Most participants were aged 30-54 (range 18-80) years	Technology or mobile health	They were provided instructions of how to conduct test, interpret the results, and posttest counseling.	N/A
Zhu et al [56], 2019	China	RCT	100	68% of the participants were aged 18-29 (range 18 to >30) years	Technology or mobile health	They were provided pretest and posttest counseling after downloading the *WeTest* mobile app, and confirmatory testing and linkage to care, and 58%-71% of the participants were satisfied the service.	N/A
Balán et al [57], 2020	United States	RCT	272	Mean 36.6	Technology or mobile health	They were provided instructions of how to use the test kits and linkage to care via daily SMS text messages.	N/A
Carballo-Diéguez et al [58], 2020	United States	RCT	272	Mean 34 (SD 11)	Technology or mobile health	They were provided rapid oral test kits, instructions, and pretest counseling.	N/A
Edelstein et al [59], 2020	United States	Cohort study	12,182	51% of the participants were aged between 25 and 34 (range 18 to >45) years	Technology or mobile health	N/A	They were only provided informational inserts developed by NYC health department with on HIV testing and pre- and post-exposure prophylaxis, and confirmatory testing and HIV care when there is a need.
Johnson et al [60], 2020	United States	Cohort study	922	70% of the participants were aged between 18 and 34 (range 18 to >45) years	Technology or mobile health	They were provided linkage to PrEP^e^ referrals, HIV/AIDS medical care, partner notification, and other prevention and supportive services.	N/A
MacGowan et al [61], 2020	United States	RCT	2655	57.3% of the participants were aged between 18 and 30 (range 18 to >30) years	Technology or mobile health	N/A	They were provided test kits but not proactively provided advice and recommendations on how to use the kits, and posttest counseling.
Okoboi et al [62], 2020	Uganda	Cross-sectional study	297	Median 25 (IQR 22-28)	Peer or community	They were provided counseling by counselors, linkage participants who tested positive for confirmatory testing and to HIV care services.	N/A
Phanuphak et al [63], 2020	Thailand	Cohort study	465	Median 26.4 (IQR 22.6-31.7)	Technology or mobile health	They were provided pretest counseling and posttest counseling.	N/A
Yan et al [64], 2020	China	Cohort study	1315	68% of the participants were aged between 15 and 40 (range 15 to >41) years	Technology or mobile health	They were provided posttest counseling, laboratory confirmation, and further treatment if they received reactive results by Kang Tong clinic in *mailing rapid test reagent kit* app.	N/A
Wang et al [65], 2020	China	Cohort study	510	Median 28 (IQR 23-36)	Peer or community	They were provided confirmatory HIV test result, received pretest and posttest counseling, and referred to treatment.	N/A
Zhang et al [66], 2020	China	RCT	230	Mean 29 (SD 7.7)	Technology or mobile health	They were provided instructions and counseling information, including 24×7 hotlines and an official WeChat study account to reach research assistants to obtain consultation on the HIVST administration and interpretation of testing results.	N/A
Zhang et al [67], 2020	China	Cross-sectional study	2364	58.5% of the participants were aged >24 years	Technology or mobile health	They were provided pretest and posttest counseling through the WeChat public platform, the individual WeChat app, or by telephone, and social media delivery strategy was faster in recruiting MSM^f^ to attend HIVST, had a higher degree of linkage to care and ART^g^, and had a lower economic cost than that of its counterpart.	N/A
Bell et al [68], 2021	Australia	Cross-sectional study	494	48% of the participants were aged between 20 and 29 years	Technology or mobile health	They were provided the pretest information, 3 monthly testing reminders via phone, email, or SMS text messaging, and a link to a posttest survey via an SMS text message, and 24% and 47% agreed with pretest counseling and posttest counseling over the phone, respectively.	N/A
Chen et al [69], 2021	South Africa	Cohort study	110	67% of the participants were aged between 18 and 24 (range 18 to >25) years	Technology or mobile health	They were provided laboratory confirmation, posttest counseling if any participant with a positive test and a care call weekly until it was confirmed the participant had linked to care or the study ended.	N/A
Cheng et al [70], 2021	China	RCT	491	90% of the participants were aged between 18 and 35 (range 18 to >36) years	Technology or mobile health	They were provided pretest counseling by consisting of a short message and reminded to do the testing at home.	N/A
Chan et al [71], 2021	China	Cohort study	350	57.1% of the participants were aged between 18 and 30 (range 18-40) years	Technology or mobile health	They were provided standard-of-care pretest counseling via video chat, web-based and real-time supervision by the administrators, and 72%-98% of the participants were satisfied the service.	N/A
Hecht et al [72], 2021	United States	Cross-sectional study	625	69% of the participants were aged between 18 and 34 (range 18 to >55) years	Technology or mobile health	They were provided posttest counseling 10 days after their HIV test kit was mailed.	N/A
Li et al [73], 2021	China	Cross-sectional study	2263	64% of the participants were aged >24 years	Technology or mobile health	They were provided HIV posttest consultation via WeChat or over the phone to help with the interpretation of the test results and referral to services for clinical confirmatory testing and antiviral treatment.	N/A
da Cruz et al [74], 2021	Brazil	Cross-sectional study	2681	Median 25 (IQR 21-30)	Technology or mobile health	They were provided linkage to HIV treatment to MSM. One-on-one and SMS text messaging were available for up to 3 months following enrollment in linkage services.	N/A
Wu et al [75], 2021	China	Cohort study	371	Mean 29 (SD 7)	Technology or mobile health	They were provided posttest counseling, including confirmatory testing and treatment services at a local health facility via telephone calls.	N/A
Zhang et al [76], 2021	China	Cohort study	471	Median 29 (IQR 25-35)	Technology or mobile health	They were provided web-based services on the application of extra testing kits, instructions on self-testing, real-time consultation with the staff, and uploading of test outcomes and posttest counseling.	N/A
Abubakari et al [77], 2021	Ghana	Cross-sectional study	61	N/A	Technology or mobile health	They were provided posttest counseling by a smartphone with a preinstalled C5 app, and more than three-quarters of the participants were satisfied the service.	N/A
Maatouk et al [78], 2021	Lebanon	Cross-sectional study	1103	Mean 26, range 18-57	Technology or mobile health	They were provided HIV counseling and guidance on testing by a hotline.	N/A
Frye et al [79], 2021	United States	RCT	111	Mean 23 (SD 4)	Peer or community	They were provided standard counseling by a counselor.	N/A
Girault et al [80], 2021	Thailand	Cross-sectional study	1422	45% aged between 15 and 24, range 15 to >30 years	Peer or community	N/A	They were provided oral instructions before and during the test, and assistance in conducting the test or interpreting the result when requested, and only 57% of the participants were satisfied with the service.
Phongphiew et al [81], 2021	Thailand	Cross-sectional study	45	Mean 17.6 (SD 1.1)	Peer or community	They were provided HIV prevention counseling and as an oral daily HIV PrEP, available in the same clinic by the same care team, and 79% of the participants preferred to do tests in hospitals rather than at home.	N/A
Widyanthini et al [82], 2021	Indonesia	Cross-sectional study	813	52% of the participants were aged between 16 and 29 (range 16 to >39) years	Peer or community	They were provided pretest and posttest counseling accordingly. If the result was reactive, the participant was referred to the HIV testing clinic at Kerti Praja Foundation Clinic for confirmatory testing.	N/A
Wirtz et al [83], 2021	Myanmar	RCT	63	Median 21 (IQR 19-25)	Peer or community	They were provided pretest counseling, posttest counseling, in-clinic point-of-care CD4 testing for immediate staging, then liked to HIV care services. Linkages to HIV care and future HIV testing for those with negative results were made to community-based affirming health facilities.	N/A
Dijkstra et al [84], 2021	Kenya, East Africa	Cross-sectional study	452	Median 26 (IQR 22-30)	Peer or community	They were provided HIV testing and counseling (HTC) with 2 rapid HIV antibody tests in series, and 77% of the participants were satisfied with the service.	N/A
O’Byrne et al [85], 2021	Canada	Cross-sectional study	111	Mean 31 years	Peer or community	They were provided pretest and posttest counseling materials and a website for ordering, which included resources and information about HIV and instructions (including videos) about self-testing, postexposure prophylaxis information, and an appointment in our nurse-led PrEP clinic (PrEP-RN).	N/A
Lillie et al [86], 2021	Burundi, East Africa	Cohort study, 364 MSM	363	Mean 27 (SD 7.6)	Peer or community	They were provided pretest and posttest counseling, supported the administration of the test, managed the screening results and ethical issues, and provided referrals for follow-up services.	N/A

^a^N/A: not applicable.

^b^RCT: randomized controlled trial.

^c^CDC: Centers for Disease Control and Prevention.

^d^HIVST: HIV self-testing.

^e^PrEP: pre-exposure prophylaxis.

^f^MSM: men who have sex with men.

^g^ART: antiretroviral therapy.

### Quality Assessment

The National Institutes of Health quality assessment tool was used to assess the quality of RCTs and observational studies [87]. The tool covers 14 domains for RCTs, observational cohorts, and cross-sectional studies, with a total score ranging from 0 to 14. Higher scores indicated better quality, and each study’s summary score was categorized as poor (0-4 out of 14 questions), fair (5-10 out of 14 questions), or good (11-14 out of 14 questions).

### Data Analysis

Meta-analyses were conducted using random effects models to combine data and calculate pooled proportions and 95% CIs based on the generalized linear mixed effects method [88]. Heterogeneity was quantified using *I*^2^ statistic. *I*^2^ values <25%, 25% to 75%, and >75% indicate low, moderate, and high heterogeneity, respectively [89]. We used visual inspection to assess the asymmetry of funnel plots and the Egger test to detect potential publication bias [90]. Sensitivity analysis was conducted by removing one study at a time.

Subgroup analyses and metaregression were conducted to assess whether the proportion of linkage to care varied and was predicted according to the values of the study characteristics. These included study year (we used 2016 as the cutoff as it was the year when the WHO started recommending HIVST) [91], study sample size (<300 vs ≥300), study countries (high income, upper middle income, lower middle income, and low income based on the new World Bank country classification) [92], HIVST counseling delivery modes (technology and mobile health vs peer and community), presence of pretest and posttest counseling (posttest counseling only vs both pre- and posttest counseling), and quality of counseling. Quality of counseling was measured by the number of essential components involved in the counseling support for MSM HIVST users.

Among all studies, we assessed whether the overall linkage to care varied by study characteristics (type of counseling support, study year, study sample size, study countries, HIVST counseling delivery modes, presence of pretest and posttest counseling, and quality of counseling) using the univariate metaregression model. Factors with *P*<.10 in univariate metaregression analyses were entered into the multivariable metaregression model. Within studies providing active or passive counseling support, univariate and multivariate metaregression were used to examine whether linkage to care varied by study characteristics. All analyses were performed with R (version 4.2.1; R Foundation for Statistical Computing) using the *metafor* package.

## Results

### Study Characteristics

**Figure 1:**
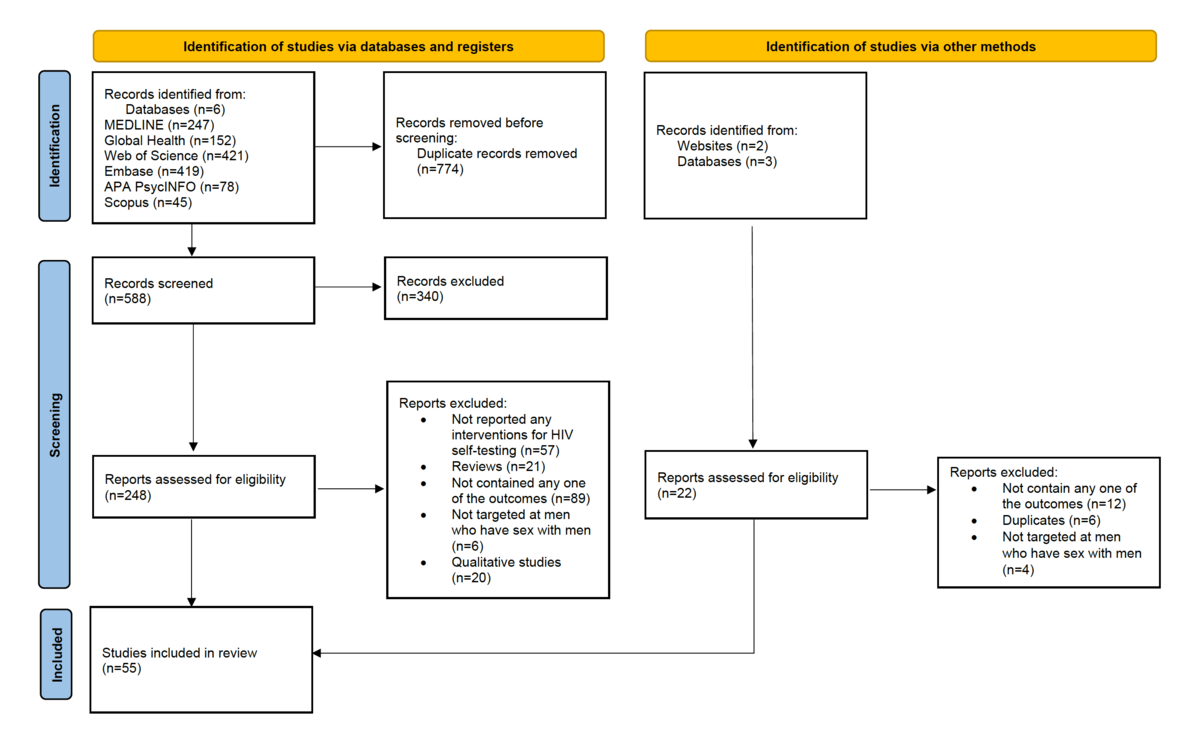
Flowchart outlining the systematic review process.

A flowchart of the literature selection process is presented in Figure 1. The initial search yielded 1362 publications through databases and registers, and 55 studies met the eligibility criteria and were included in the systematic review [27,33-86]. All 55 studies were included in the meta-analysis to estimate the pooled proportion of linkage to care among MSM HIVST users, categorized by active and passive counseling support.

The characteristics of the included studies are presented in Table 2, and the results for studies that assessed linkage to care are presented in Table 3. All studies were published from 2014 to 2021, including 53 full-text manuscripts [27,33,34,36-51,53-86] and 2 conference abstracts [35,52]. The 55 included studies reported data on 45,147 MSM aged 15 to 73 years. According to the geographical location, 26 (47%) studies were conducted in the Asia-Pacific region, 16 (29%) in North America, 9 (16%) in Africa, 3 (6%) in South America, and 1 (2%) in Europe. Of the included studies, based on the study design, 43 (78%) were observational studies, including 28 (65%) cross-sectional and 15 (35%) cohort studies, and 12 (22%) were RCTs. Detailed results of the quality assessment of the studies are presented in Multimedia Appendix 3 [27,33-86].

**Table 3:** Results for studies that assessed linkage to care.

Linkage to care		Overall proportion, (95% CI)	*I^2^*	Egger test (*P* value)	Number of estimates (references)
**Active counseling support**					
	Reporting test results	97.2 (74.3%-99.8%)	100%	.25	9 [41,51,56,64,66,67,73,75,76]
	Laboratory confirmation	92.6 (86.1%-96.2%)	57%	.34	35 [34,36,37,39-47,49,51-53,56,58,60,62,63,65,66,68,69,71,73-75,78,79,81-83,86]
	ART^a^ initiation	90.8 (86.7%-93.7%)	0%	.97	21 [37,43-45,47,49,52,53,60,62,63,66,67,70,71,73,78,81,82,84,86]
	Referral to physicians	96.3 (85%-99.2%)	0%	.14	28 [33,35,36,38,39,41,44-47,49,54,55-57,60,63,65,66,68-71,74,75,77,79,81]
	Prevention strategies	100.0 (0%-100%)	0%	<.001	7 [34,49,60,63,71,81,85]
	PrEP^b^ initiation	27.0 (10.2%-54.6%)	97%	.73	6 [48,60,72,81,84,85]
**Passive** **counseling support**					
	Reporting test results	—^c^	—	—	1 [50]
	Laboratory confirmation	78.7 (17.8%-98.4%)	82%	.02	5 [27,50,59,61,80]
	ART initiation	79.1 (48.8%-93.7%)	0%	.06	4 [50,59,61,80]
	Referral to physician	79.1 (0%-100%)	91%	<.001	2 [59,61]
	Prevention strategies	—	—	—	—
	PrEP initiation	—	—	—	—

^a^ART: antitretroviral therapy.

^b^PrEP: pre-exposure prophylaxis.

^c^Pooled proportion was not performed because of fewer than 2 studies.

### Active Counseling Supporting HIVST

Overall, 91% (50/55) of the studies provided active counseling support for MSM HIVST users [33-49,51-58,60,62-79,81-86]. These studies were conducted in China (14/50, 28%), the United States (12/50, 24%), Brazil (3/50, 6%), Australia (2/50, 4%), Uganda (2/50, 4%), Vietnam (2/50, 4%), South Africa (2/50, 4%), Canada (2/50, 4%), Burundi (2/50, 4%), Thailand (2/50, 4%), the United Kingdom (1/50, 2%), Nigeria (1/50, 2%), Ghana (1/50, 2%), Lebanon (1/50, 2%), Indonesia (1/50, 2%), Myanmar (1/50, 2%), and Kenya (1/50, 2%). Most of these studies (38/50, 76%) were conducted in high-income and upper middle–income countries.

Moreover, 66% (33/50) of the studies used mobile health technology to deliver active counseling support [33,34,36-39,41,45,46,48,49,51,54-58,60,63,64,66-78]. Mobile phones and the internet (eg, telephone calls, SMS text messages, and emails) were the most commonly used technology (21/33, 64%), followed by social media apps or geospatial dating apps (eg, WeChat, QQ, Blued, and Grindr; 8/33, 24%) and HIVST-specific apps (eg, HIVSmart!, WeTest, and C5 apps; 4/33, 12%). Other studies (n=17) used sex partners (3/17, 18%) and nurses or physicians (14/17, 82%) to deliver [33,38,42,44,45,52,55,56,64,76-83].

### Essential Components Involved in the Pretest Counseling of the Active Counseling Supporting HIVST

In the pretest counseling, 32 studies proactively provided at least one essential component to MSM HIVST users (Multimedia Appendix 4 [27,33-86]). Five studies provided only one essential component, such as informing users of the benefits of taking the tests (1/5, 20%), assuring users’ rights to refuse HIV testing (2/5, 40%), or providing HIV prevention information (2/5, 40%). Five other studies provided 2 essential components. In addition to informing users of the benefits of taking the tests, these studies provided risk assessment (1/5, 20%), assured users’ right to refuse (2/5, 40%), encouraged beneficial disclosure of HIV serostatus (1/5, 20%), or provided HIV prevention information (1/5, 20%). Four other studies provided 3 essential components. In addition to informing the users about the benefits of taking the test, the combination of other components were (1) providing risk assessment and HIV prevention information (1/4, 25%), (2) encouraging beneficial disclosure of serostatus and providing HIV prevention information (1/4, 25%), (3) assuring users’ right to refuse HIV testing and providing HIV prevention information (1/4, 25%), and (4) providing risk assessment and assuring users’ right to refuse (1/4, 25%). Another study provided 4 essential components: (1) informing users of the benefits of taking the tests, (2) providing risk assessment, (3) assuring the user’s right to refuse HIV testing, and (4) encouraging beneficial disclosure of serostatus status. The remaining studies (n=13) provided all 5 essential components.

In addition to these essential components, 15 studies provided other supplementary components. These components consisted of (1) the reason for HIVST (1/15, 7%), (2) stories addressing general health concerns of MSM (2/15, 13%), (3) the assessment of potential social support (4/15, 27%), (4) first-person stories about people living with HIV (1/15, 7%), and (5) local data, news, and policies regarding HIV and sexually transmitted infections among MSM (7/15, 47%).

### Essential Components Involved in the Posttest Counseling of the Active Counseling Supporting HIVST

In posttest counseling, 50 studies proactively provided at least one essential component to MSM HIVST users (Multimedia Appendix 4). Six studies only provided one essential component, offering additional HIV care to MSM who received reactive HIVST results. Moreover, 24 studies provided 2 essential components. These combinations included (1) additional HIV care for users with reactive HIVST results and interpretation of HIVST results (23/24, 96%) and (2) additional HIV care for users with reactive HIVST results and HIV prevention information for users with negative HIVST results (1/24, 4%). The rest of the studies (n=18) provided all 3 essential components (eg, interpretation of testing results, HIV prevention information for users with negative HIVST results, and additional HIV care for users with reactive HIVST results).

### Passive Counseling Supporting HIVST

Passive counseling support to MSM was provided in 10% (5/50) of the studies [27,50,59,61,80]. These studies were conducted in the United States (2/5, 40%), China (1/5, 20%), Indonesia (1/5, 20%), and Thailand (1/5, 20%). Three studies used mobile health technology to deliver counseling [50,59,61], whereas the other 2 studies used peers and communities to provide counseling [27,80].

### Essential Components Involved in the Pretest Counseling of Passive Counseling Supporting HIVST

In the pretest counseling, 3 studies offered at least one essential component upon request. One study only informed the benefits of taking the tests. The other 2 studies included 2 essential components: informing the participants of the benefits of taking the tests and providing HIV prevention information. In addition to these essential components, one study provided local data, news, and policies regarding HIV and sexually transmitted infections among MSM.

### Essential Components Involved in the Posttest Counseling of the Passive Counseling Supporting HIVST

In posttest counseling, all 5 studies provided at least one essential component upon request. Three studies provided one essential component, such as interpretation of the HIVST results (1/5, 20%), or provision of psychological support and referral to HIV care for users with positive results (2/5, 40%). The other 2 studies provided 2 essential components. In addition to the interpretation of HIVST results, these studies provided referral to HIV care for users with positive results (1/5, 20%) or HIV prevention information for users with negative results (Multimedia Appendix 4).

### Meta-Analysis of Linkage to Care Among MSM HIVST Users Along With Active and Passive Counseling

**Figure 2:**
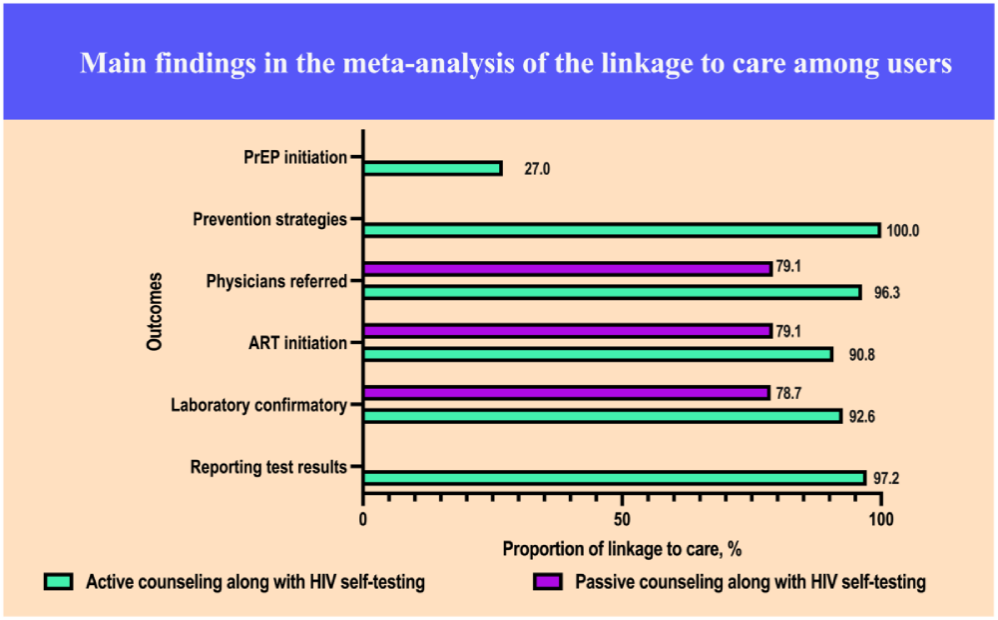
Main findings in the meta-analysis of the linkage to care among users. ART: antiretroviral therapy; PrEP: pre-exposure prophylaxis.

The main findings of the meta-analysis of the linkage to care among MSM HIVST users are summarized in Figure 2.

### Reporting Testing Results

**Figure 3:**
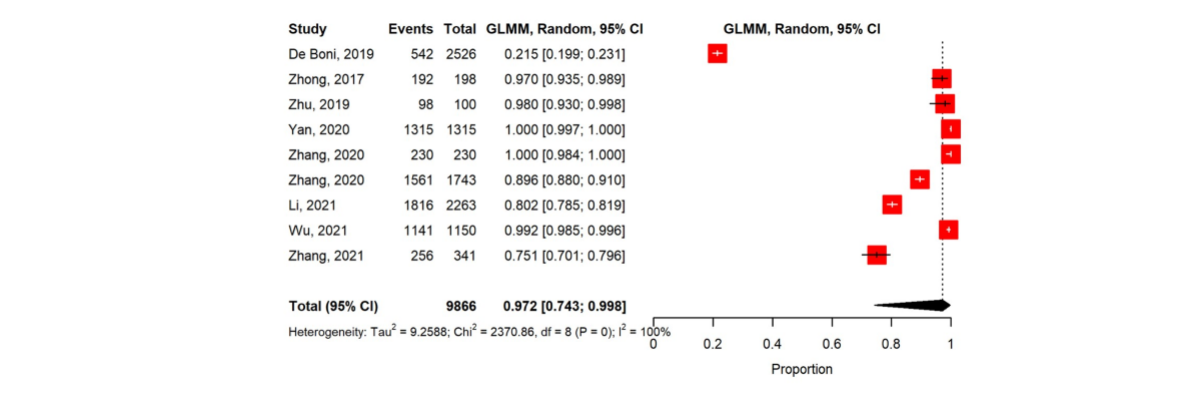
Forest plot of the pooled proportion of reporting HIV self-testing results.

Our meta-analysis comprised 45,147 MSM, using a random effects model (Table 3). Overall, 10 studies measured the proportion of users who reported HIVST results [41,50,51,56,64,66,67,73,75,76]. In studies providing active counseling support, the pooled proportion of reporting HIVST results was 97.2% (n=9; 95% CI 74.3%-99.8%; *I*^2^=100%; Figure 3 [41,51,56,64,66,67,73,75,76]). Only one study providing passive counseling support reported a proportion of 77.7% for reporting HIVST results [50]. However, this study was not included in the meta-analysis owing to insufficient data.

### Linkage to Laboratory Confirmation

**Figure 4:**
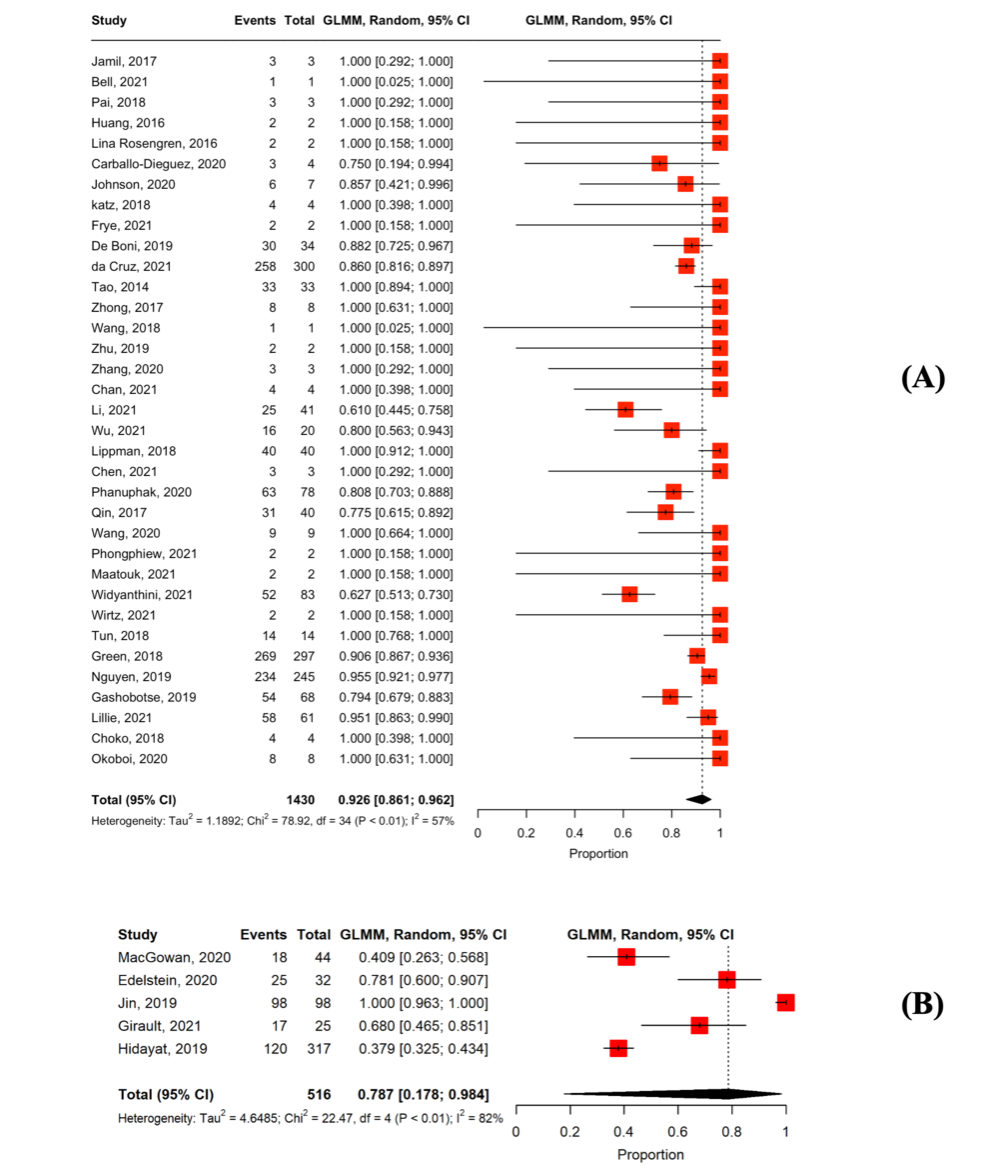
Forest plot of the pooled proportion of linkage to laboratory confirmation among users with reactive results: (A) studies were provided active counseling along with HIV self-testing and (B) studies were provided passive counseling along with HIV self-testing.

In total, 40 studies assessed linkage to laboratory confirmation among users with positive results [27,34,36,37,39-47,49-53,56,58-63,65,66,68,69,71,73-75,78-83,86]. In the studies providing active counseling support, the pooled proportion of linkage to laboratory confirmation was 92.6% (n=35; 95% CI 86.1%-96.2%; *I*^2^=57%; Figure 4 [34,36,37,39-47,49,51-53,56,58,60,62,63,65,66,68,69,71,73-75,78,79,81-83,86]). In studies providing passive counseling support, the pooled proportion of linkage to laboratory confirmation was 78.7% (n=5; 95% CI 17.8%-98.4%; *I*^2^=82%; Figure 4).

### Linkage to ART Initiation

**Figure 5:**
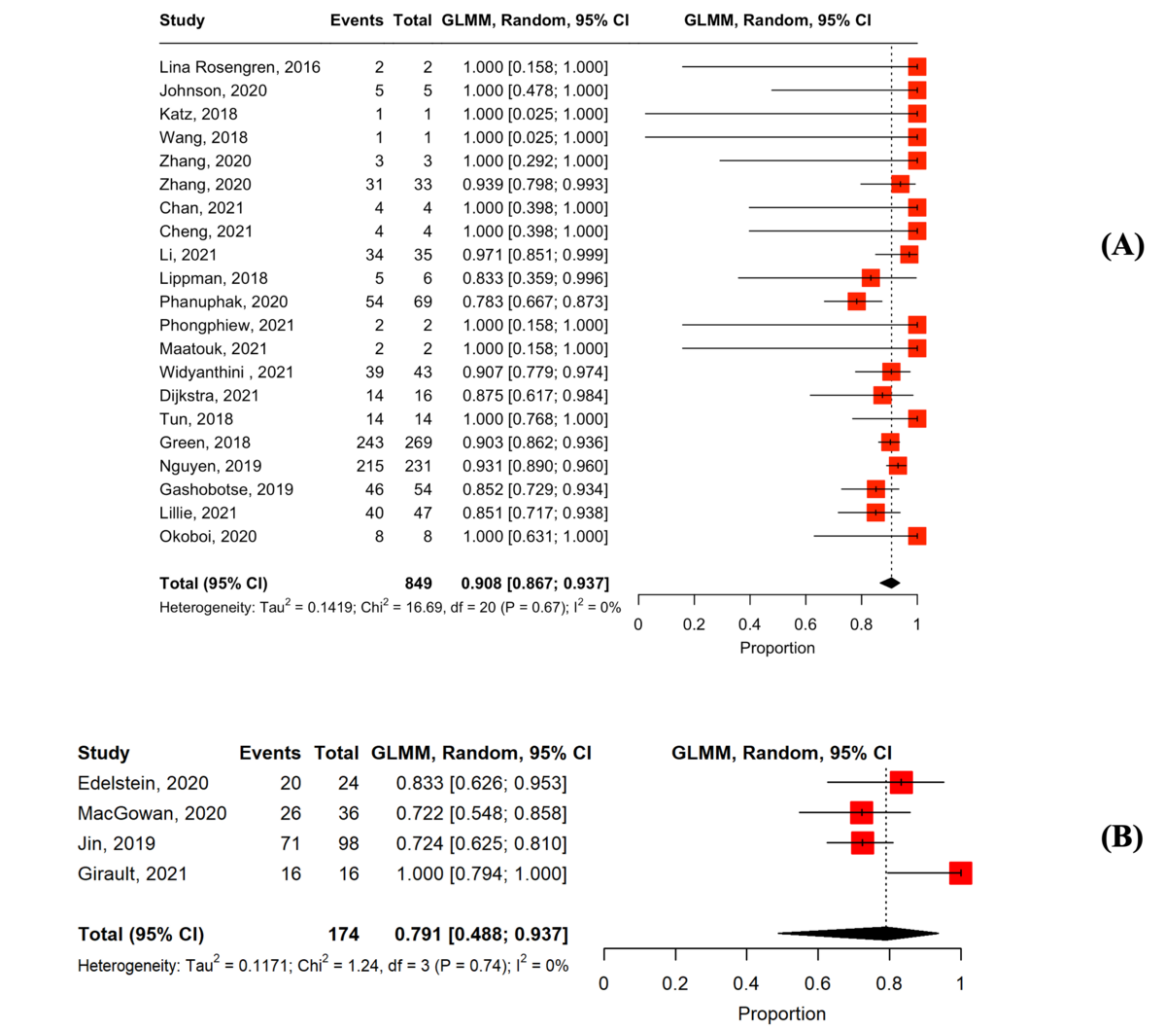
Forest plot of the pooled proportion of linkage to antiretroviral therapy among users who were confirmed HIV positive: (A) Studies were provided active counseling along with HIV self-testing and (B) studies were provided passive counseling along with HIV self-testing.

Overall, 25 studies reported linkage to ART initiation among users with positive results [37,43-45,47,49,50,52,53,59-63,66,67,70,71,73,78,80-82,84,86]. In the studies providing active counseling support, the pooled proportion of ART initiation was 90.8% (n=21; 95% CI 86.7%-93.7%; *I*^2^=0%; Figure 5 [37,43-45,47,49,52,53,60,62,63,66,67,70,71,73,78,81,82,84,86]). In studies with passive counseling support, the pooled proportion of ART initiation was 79.1% (n=4; 95% CI 48.8%-93.7%; *I*^2^=0%; Figure 5) [50,59,61,80].

### Linkage to Physicians

**Figure 6:**
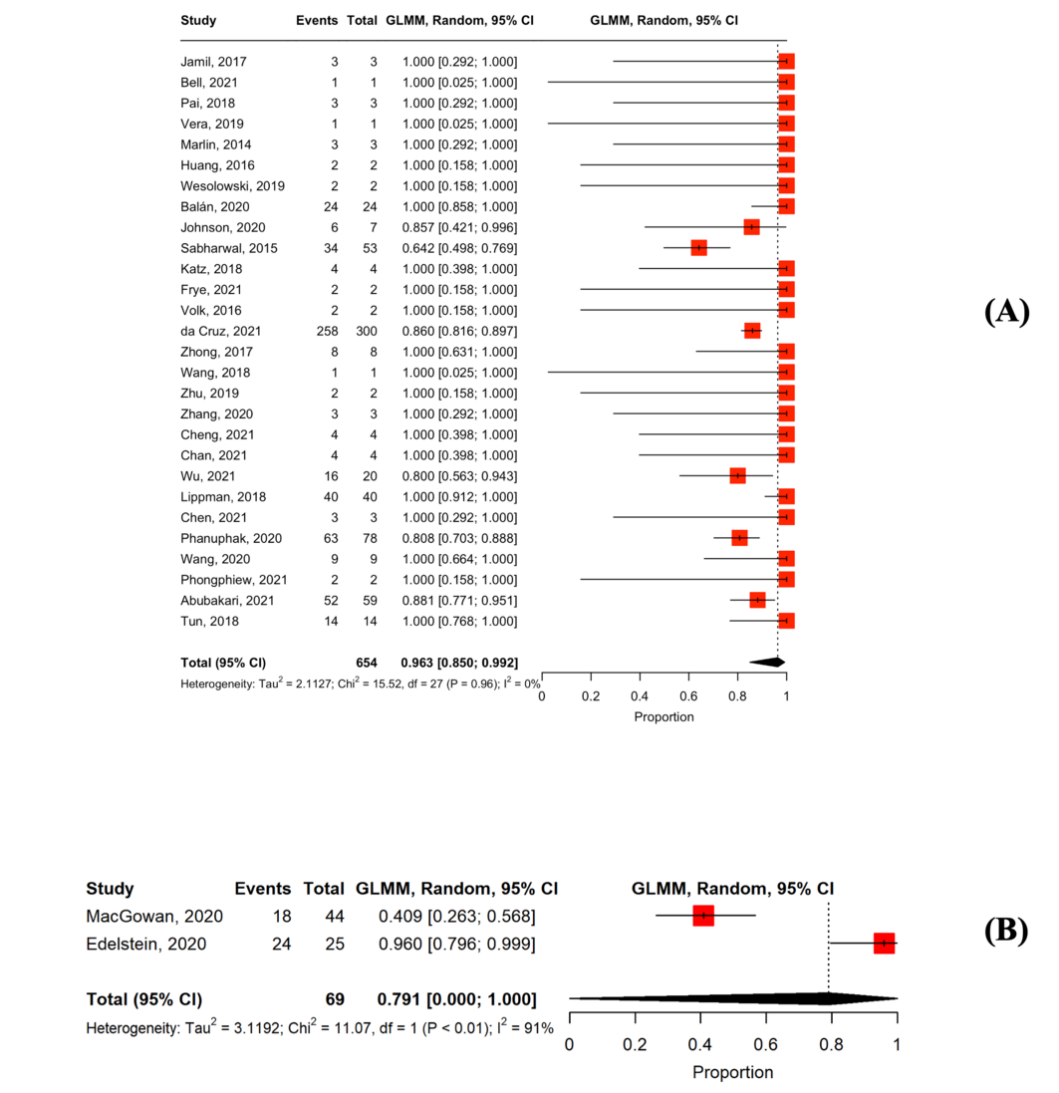
Forest plot of the pooled proportion of linkage to physicians among users with reactive results: (A) studies were provided active counseling along with HIV self-testing and (B) studies were provided passive counseling along with HIV self-testing.

In total, 30 studies reported linkage to physicians among users with positive results [33,35,36,38,39,41,44-47,49,54,55-57,59-61,63,65,66,68-71,74,75,77,79,81]. In the studies providing active counseling support, the pooled proportion of linkage to physicians was 96.3% (n=28; 95% CI 85%-99.2%; *I*^2^=0%; Figure 6 [33,35,36,38,39,41,44-47,49,54,55-57,60,63,65,66,68-71,74,,75,77,79,81]). In the studies with passive counseling support, the pooled proportion of linkage to physicians was 79.1% (n=2; 95% CI 0%-100%; *I*^2^=91%; Figure 6) [59,61].

### Linkage to Information Related to Sexual Risk Behaviors Reduction and PrEP and PrEP Initiation

**Figure 7:**
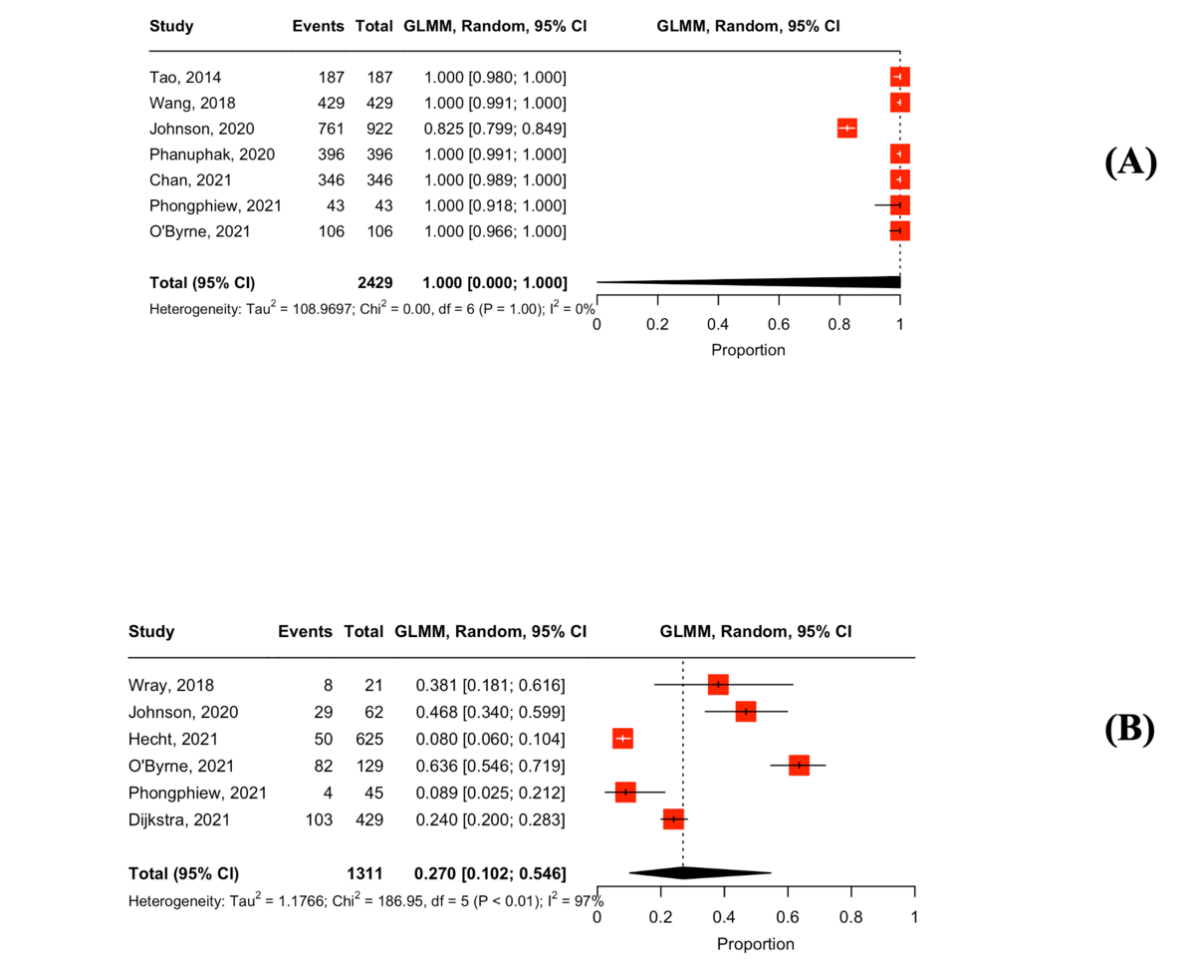
Forest plot of the (A) pooled proportion of linkage to information related to sexual risk behaviors reduction and pre-exposure prophylaxis (PrEP) among users with negative results and (B) pooled proportion of PrEP initiation among users with negative results.

Overall, 7 studies reported linkage to information related to sexual risk behavior reduction and PrEP among users with negative results. In studies with active counseling support, the pooled proportion of linkage to sexual risk behaviors reduction was 100% (n=7; 95% CI 0%-100%; *I*^2^=0%; Figure 7 [34,49,60,63,71,81,85]). The pooled proportion of PrEP initiation was 27% (n=6; 95% CI 10.2%-54.6%; *I*^2^=97%; Figure 7) [48,60,72,81,84,85] in studies providing active counseling support. No studies with passive counseling support reported a linkage to information related to sexual risk behaviors and PrEP or PrEP initiation among users with negative results.

### Publication Bias

**Figure 8:**
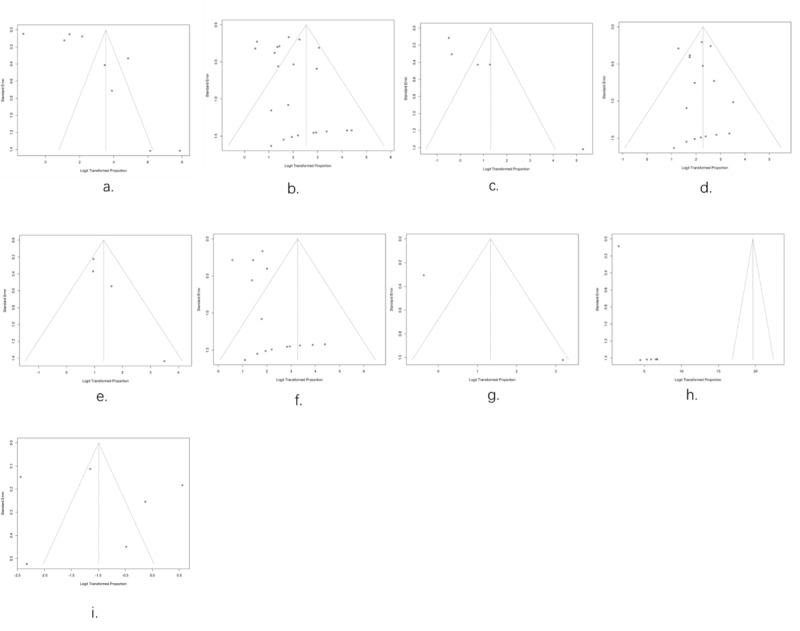
Funnel plots for assessing the publication bias among the included studies: (A) linkage to reporting test results; (B) linkage to laboratory confirmation with active counseling support; (C) linkage to laboratory confirmation with passive counseling support; (D) linkage to antiretroviral therapy (ART) initiation with active counseling support; (E) linkage to ART initiation with passive counseling support; (F) linkage to physicians with active counseling support; (G) linkage to physicians with passive counseling support; (H) linkage to information related to sexual risk behaviors reduction, and pre-exposure prophylaxis (PrEP); and (I) linkage to PrEP initiation.

Upon examination of the funnel plots (Figure 8; Table 3), there was a publication bias in studies reporting the proportion of linkage to laboratory confirmation (*P*=.02), referral to physicians (*P*<.001), and prevention strategies (*P*<.001). Furthermore, outliers were identified in studies reporting the proportion of reporting test results [51] and linkage to laboratory confirmation [73,82], ART initiation [63], physicians [35], and PrEP initiation [85] (Figure 8).

### Sensitivity Analysis

For studies providing active counseling support, the pooled proportion of reporting test results, and linkage to laboratory confirmation, ART initiation, physicians, information related to sexual risk behaviors reduction and PrEP, and PrEP initiation changed slightly after removing one study at each time.

With regard to the studies providing passive counseling support, the pooled proportion of linkage to ART initiation did not change after removing one study at each time. However, after removing the study conducted by Jin et al [50], the pooled proportion of linkage to laboratory confirmation changed from 78.7% (95% CI 17.8%-98.4%) to 55.5% (95% CI 27%-80.8%). As there were only 2 studies with passive counseling support that reported linkage to physicians, a sensitivity analysis was not conducted. Details of the sensitivity analysis results are presented in Multimedia Appendix 5 [27,33-86].

### Subgroup Analysis and Metaregression

Subgroup analysis by study sample size (<300 vs ≥300) revealed different levels of linkage to reporting test results (98.5% vs 82.7%), laboratory confirmation (93.8% vs 85.6%), physicians (92% vs 86%), and PrEP initiation (35.6% vs 23.9%). The subsequent subgroup analysis (posttest counseling only vs both pre- and posttest counseling) also found different proportions of users who reported test results (87.1% vs 93.2%) and initiated PrEP (20.2% vs 53%). Subgroup analysis by other study characteristics did not reveal a large difference in the linkage to care.

Among all studies, univariate metaregression analysis demonstrated that the type of counseling (active vs passive), a smaller sample size (<300 vs ≥300), and a higher number of essential components involved in the counseling support were significantly associated with better linkage to care. Furthermore, the multivariate metaregression analysis confirmed that a larger sample size was linked to a lower linkage to care (*P*=.03). In contrast, mobile health technology counseling (*P*=.05) and a higher number of essential components involved in the counseling support were associated with increased linkage to care (*P*=.04).

In studies providing active counseling support, univariate metaregression analysis indicated a smaller sample size (<300 vs ≥300), provision of both pretest and posttest counseling (vs posttest counseling only), and a higher number of essential components were significantly associated with better linkage to care. The findings of multivariate metaregression analysis revealed that a smaller sample size (*P*=.03) and using mobile health technology for counseling (*P*=.05) were associated with a higher linkage to care. With regard to different outcomes related to linkage to care, a larger sample size was correlated with a lower linkage to laboratory confirmation (*P*=.03) and prevention strategies (*P*<.001) with active counseling support (Multimedia Appendix 6).

## Discussion

### Principal Findings

This systematic review and meta-analysis aimed to summarize the global evidence on counseling support and synthesize the proportion of linkage to care among MSM HIVST users. We categorized counseling support in assisted HIVST into active or passive. More than 90% of the MSM HIVST users with reactive results were linked to laboratory confirmation and ART initiation in studies implementing active counseling support. Such a proportion was higher than that of the studies with passive counseling support (78.7%-79.1%). Therefore, the provision of active counseling support may be helpful in improving the linkage to HIV care and treatment for MSM HIVST users with reactive results.

Relatively few studies (7/55, 13%) provided information related to sexual risk behaviors reduction and PrEP for MSM HIVST users with negative results. One possible explanation was that most resources were used to provide support for users with reactive results, which was considered a priority for some HIVST programs [93]. Hence, there are constraints in resources to provide support for the large number of users with negative results [93]. In addition to identifying individuals testing positive for HIV, facilitating behavior changes is an important purpose of HIV testing and counseling. Future studies should consider providing more comprehensive support for MSM HIVST users with negative results.

The metaregression results identified some significant determinants of the linkage to care among MSM HIVST users. First, more essential counseling components were associated with better linkage to care, which aligns with findings from a previous study [94]. Incorporating a higher number of essential components would enhance counseling quality. Previous studies suggest that delivering high-quality counseling improves linkage to care, reduces risky behaviors, and prevents new infections [95,96]. However, our study found that only 26% to 36% of the studies provided all essential active pretest and posttest counseling support. As a result, future programs should consider offering comprehensive counseling to MSM HIVST users.

Second, a larger sample size was associated with a lower linkage to care among MSM HIVST users. Providing active counseling support for HIVST users was resource demanding. For example, it took 1 hour to prepare and implement one session of real-time pretest and posttest counseling support for each MSM HIVST user [49,71]. Therefore, it is challenging to provide counseling support to a larger number of HIVST users.

Furthermore, mobile health technology counseling was associated with a better linkage to care compared with peer and community counseling. Our study found that mobile health technology counseling is the predominant method to support MSM HIVST users, which aligns with a previous study [28]. Owing to high smartphone ownership among MSM (>94%) [97], mobile health technology presents a viable strategy for counseling among MSM HIVST users. In addition, previous studies have demonstrated that using mobile health technology for counseling support reduces the workload of HIV testing administrators [28,98].

Stigma and discrimination against MSM impede access to HIV testing and counseling services [99]. Systematic reviews have shown that perceived stigma remained a significant obstacle to engaging in assisted HIVST [100,101]. Future programs should consider increasing the empathy of health workers who provide counseling to support HIVST. A previous study suggested that the negative effects of perceived stigma or discrimination on HIV testing use could be offset by increasing the empathy of service providers [102]. A recent study applied computerized programs (instead of people) to provide active counseling supporting HIVST [98]. Such an approach could also reduce the concerns of stigma or discrimination when using assisted HIVST among MSM.

### Limitations

There are several limitations in this study. First, there was high heterogeneity among the studies that reported outcomes on linkage to laboratory confirmation, physicians with the provision of passive counseling support, linkage to PrEP initiation, and reporting test results. Heterogeneity pertains to the diversity observed in the design of studies, the effects of interventions, or the outcomes obtained across different studies. Persistent heterogeneity could not be resolved using sensitivity analysis. Second, publication bias was found in relation to the pooled proportion of linkage to laboratory confirmation, physicians, and prevention strategies, which could impact the validity and generalization of conclusions. Third, because half of the included studies were cross-sectional, a causal relationship could not be established. Furthermore, the use of nonprobabilistic sampling for MSM in all the included studies limited the generalizability of the findings. Finally, the small number of studies in the comparison group (those providing passive counseling) would result in bias when comparing the study outcomes between active and passive counseling support.

### Conclusions

This study synthesized evidence on active and passive counseling support for MSM HIVST users and quantified the proportion of linkage to care. As compared with passive counseling support, active counseling support had a better linkage to care. Having a higher number of essential counseling components, a smaller sample size, and using mobile health technology to deliver counseling support were also associated with a better linkage to care. As our results showed, proactively providing counseling support for all users, involving more essential components in the counseling support, and using mobile health technology should be considered to increase the linkage to care among MSM HIVST users.

## Appendix

Multimedia Appendix 1 PRISMA (Preferred Reporting Items for Systematic Reviews and Meta-Analyses) checklist.

Multimedia Appendix 2 Search terms.

Multimedia Appendix 3 Quality assessment of the included studies using the National Institutes of Health quality assessment tool.

Multimedia Appendix 4 Summary of counseling components in included studies.

Multimedia Appendix 5 Summary of sensitivity analysis.

Multimedia Appendix 6 Subgroup analysis and metaregression analyses.

## Abbreviations

ART: antiretroviral therapy
HIVST: HIV self-testing
MSM: men who have sex with men
PrEP: pre-exposure prophylaxis
PRISMA: Preferred Reporting Items for Systematic Reviews and Meta-Analyses
RCT: randomized controlled trial
UNAIDS: Joint United Nations Programme on HIV/AIDS
WHO: World Health Organization

## Data Availability

All data generated or analyzed during this study are included in this published paper (and its Multimedia Appendix files).
