# Supplementary material for: Counseling Supporting HIV Self-Testing and Linkage to Care Among Men Who Have Sex With Men: Systematic Review and Meta-Analysis
Source: JMIR Public Health Surveill. 2024 Jan 24;10:e45647. doi: 10.2196/45647 (PMC10851126; doi:10.2196/45647)
Supplement: Multimedia Appendix 1 [file publichealth_v10i1e45647_app1.docx]

# Multimedia Appendix 1. PRISMA Checklist

| **Section and Topic** | **Item #** | **Checklist item** | **Location where item is reported** |
| --- | --- | --- | --- |
| **TITLE** | | |  |
| Title | 1 | Identify the report as a systematic review. | Title, Cover page |
| **ABSTRACT** | | |  |
| Abstract | 2 | See the PRISMA 2020 for Abstracts checklist. | Abstract |
| **INTRODUCTION** | | |  |
| Rationale | 3 | Describe the rationale for the review in the context of existing knowledge. | Introduction, Paragraph 1-5 |
| Objectives | 4 | Provide an explicit statement of the objective(s) or question(s) the review addresses. | Introduction, Paragraph 5 |
| **METHODS** | | |  |
| Eligibility criteria | 5 | Specify the inclusion and exclusion criteria for the review and how studies were grouped for the syntheses. | Methods – Inclusion and exclusion criteria, Paragraph 1 and Table 1  Data analysis, Paragraph 1 |
| Information sources | 6 | Specify all databases, registers, websites, organisations, reference lists and other sources searched or consulted to identify studies. Specify the date when each source was last searched or consulted. | Methods - Search strategy, Paragraph 1  Methods - Search strategy, Paragraph 2 |
| Search strategy | 7 | Present the full search strategies for all databases, registers and websites, including any filters and limits used. | Methods – Search strategy, Paragraph 1  Search strategies –Multimedia Appendix 2 for details regarding search strategies |
| Selection process | 8 | Specify the methods used to decide whether a study met the inclusion criteria of the review, including how many reviewers screened each record and each report retrieved, whether they worked independently, and if applicable, details of automation tools used in the process. | Methods – Data extraction, Paragraph 1 |
| Data collection process | 9 | Specify the methods used to collect data from reports, including how many reviewers collected data from each report, whether they worked independently, any processes for obtaining or confirming data from study investigators, and if applicable, details of automation tools used in the process. | Methods – Data extraction, Paragraph 1 |
| Data items | 10a | List and define all outcomes for which data were sought. Specify whether all results that were compatible with each outcome domain in each study were sought (e.g. for all measures, time points, analyses), and if not, the methods used to decide which results to collect. | Methods – Data extraction, Paragraph 1  Characteristics, active counseling, and passive counseling support of included studies – Table 2 |
|  | 10b | List and define all other variables for which data were sought (e.g. participant and intervention characteristics, funding sources). Describe any assumptions made about any missing or unclear information. | Methods – Data extraction, Paragraph 1  Exposure categories and outcomes included in the review of linkage to care following HIV self-testing (HIVST), along with counseling – Texbox 1 |
| Study risk of bias assessment | 11 | Specify the methods used to assess risk of bias in the included studies, including details of the tool(s) used, how many reviewers assessed each study and whether they worked independently, and if applicable, details of automation tools used in the process. | Methods – Quality assessment, Paragraph 1 |
| Effect measures | 12 | Specify for each outcome the effect measure(s) (e.g. risk ratio, mean difference) used in the synthesis or presentation of results. | Methods – Data analysis, Paragraph 1 |
| Synthesis methods | 13a | Describe the processes used to decide which studies were eligible for each synthesis (e.g. tabulating the study intervention characteristics and comparing against the planned groups for each synthesis (item #5). | Methods- Data analysis, Paragraph 1 |
|  | 13b | Describe any methods required to prepare the data for presentation or synthesis, such as handling of missing summary statistics, or data conversions. | Methods- Data analysis |
|  | 13c | Describe any methods used to tabulate or visually display results of individual studies and syntheses. | Methods- Data analysis |
|  | 13d | Describe any methods used to synthesize results and provide a rationale for the choice(s). If meta-analysis was performed, describe the model(s), method(s) to identify the presence and extent of statistical heterogeneity, and software package(s) used. | Methods- data analysis  Meta-analyses were conducted using random-effects models to combine data and calculated pooled proportions and 95% confidence intervals (CI) based on the generalized linear mixed-effects method.21 Heterogeneity was quantified using the *I*^2^ statistic. *I*^2^ values of <25%, 25 to 75%, and >75% indicate low, moderate, and high heterogeneity, respectively. We used visual inspection to assess the asymmetry of funnel plots and the Egger test to detect potential publication bias. |
|  | 13e | Describe any methods used to explore possible causes of heterogeneity among study results (e.g. subgroup analysis, meta-regression). | Methods- Data analysis, Paragraphs 2  Methods- Data analysis, Paragraphs 3 |
|  | 13f | Describe any sensitivity analyses conducted to assess robustness of the synthesized results. | Methods- Data analysis, Paragraph 1 |
| Reporting bias assessment | 14 | Describe any methods used to assess risk of bias due to missing results in a synthesis (arising from reporting biases). | Methods- Data analysis, Paragraph 1 |
| Certainty assessment | 15 | Describe any methods used to assess certainty (or confidence) in the body of evidence for an outcome. | Methods- Data analysis, Paragraph 1  Methods- Data analysis, Paragraphs 2  Methods- Data analysis, Paragraphs 3 |
| **RESULTS** | | |  |
| Study selection | 16a | Describe the results of the search and selection process, from the number of records identified in the search to the number of studies included in the review, ideally using a flow diagram. | Results – Study characteristics, Paragraph 1  Results – Study characteristics, Paragraph 2 |
|  | 16b | Cite studies that might appear to meet the inclusion criteria, but which were excluded, and explain why they were excluded. | Results – Study characteristics, Paragraph 1  Results – Study characteristics, Paragraph 2 |
| Study characteristics | 17 | Cite each included study and present its characteristics. | Results for studies that assessed linkage to care – Table 3 |
| Risk of bias in studies | 18 | Present assessments of risk of bias for each included study. | Studies were assessed using the National Institute of Health (NIH) quality assessment tool for controlled intervention studies –Multimedia Appendix 3 regarding quality assessment tool for included studies  Studies were assessed using the National Institute of Health (NIH) quality assessment tool for observational cohort and cross-sectional studies Multimedia Appendix 3 regarding quality assessment tool for included studies |
| Results of individual studies | 19 | For all outcomes, present, for each study: (a) summary statistics for each group (where appropriate) and (b) an effect estimate and its precision (e.g. confidence/credible interval), ideally using structured tables or plots. | Results, Table 2-3, Multimedia Appendix 4 and 5  Tables 2-3.  Multimedia Appendix 4: Summary of active counseling support in included studies  Multimedia Appendix 4: Summary of passive counseling support in included studies  Multimedia Appendix 5: Summary of sensitivity analysis  Multimedia Appendix 6: Univariate and Multivariable Meta-regression Analyses of Linkage to care by Study Characteristics  Multimedia Appendix 6: Sub-group Meta-analysis and Meta-regression Analyses of Linkage to Care by Study Characteristics |
| Results of syntheses | 20a | For each synthesis, briefly summarise the characteristics and risk of bias among contributing studies. | Results – Study characteristics, Paragraph 1  Results – Study characteristics, Paragraph 2 |
|  | 20b | Present results of all statistical syntheses conducted. If meta-analysis was done, present for each the summary estimate and its precision (e.g. confidence/credible interval) and measures of statistical heterogeneity. If comparing groups, describe the direction of the effect. | Abstract  Results –Meta-analysis of linkage to care among MSM HIVST users along with active and passive counseling, Reporting testing results  Results –Meta-analysis of linkage to care among MSM HIVST users along with active and passive counseling, Laboratory confirmation  Results –Meta-analysis of linkage to care among MSM HIVST users along with active and passive counseling, ART initiation  Results –Meta-analysis of linkage to care among MSM HIVST users along with active and passive counseling, Referred to physicians  Results –Meta-analysis of linkage to care among MSM HIVST users along with active and passive counseling, Linkage to Information Related to Sexual Risk Behaviors Reduction and PrEP and PrEP Initiation  Results –Meta-analysis of linkage to care among MSM HIVST users along with active and passive counseling, Publication bias  Results –Meta-analysis of linkage to care among MSM HIVST users along with active and passive counseling, Sensitivity analysis  Results –Meta-analysis of linkage to care among MSM HIVST users along with active and passive counseling, Sub-group Analysis and Meta-regression |
|  | 20c | Present results of all investigations of possible causes of heterogeneity among study results. | Figure 1  Figure 2  Figure 3  Figure 4  Figure 5  Figure 6  Figure 7  Results –Meta-analysis of linkage to care among MSM HIVST users along with active and passive counseling |
|  | 20d | Present results of all sensitivity analyses conducted to assess the robustness of the synthesized results. | Results –Meta-analysis of linkage to care among MSM HIVST users along with active and passive counseling, Sensitivity analysis |
| Reporting biases | 21 | Present assessments of risk of bias due to missing results (arising from reporting biases) for each synthesis assessed. | Figure 8 |
| Certainty of evidence | 22 | Present assessments of certainty (or confidence) in the body of evidence for each outcome assessed. | Abstract  Results –Meta-analysis of linkage to care among MSM HIVST users along with active and passive counseling, Reporting testing results  Results –Meta-analysis of linkage to care among MSM HIVST users along with active and passive counseling, Laboratory confirmation  Results –Meta-analysis of linkage to care among MSM HIVST users along with active and passive counseling, ART initiation  Results –Meta-analysis of linkage to care among MSM HIVST users along with active and passive counseling, Referred to physicians  Results –Meta-analysis of linkage to care among MSM HIVST users along with active and passive counseling, Linkage to Information Related to Sexual Risk Behaviors Reduction and PrEP and PrEP Initiation  Results –Meta-analysis of linkage to care among MSM HIVST users along with active and passive counseling, Publication bias  Results –Meta-analysis of linkage to care among MSM HIVST users along with active and passive counseling, Sensitivity analysis  Results –Meta-analysis of linkage to care among MSM HIVST users along with active and passive counseling, Sub-group Analysis and Meta-regression |
| **DISCUSSION** | | |  |
| Discussion | 23a | Provide a general interpretation of the results in the context of other evidence. | Discussion, principal findings |
|  | 23b | Discuss any limitations of the evidence included in the review. | Discussion, limitations |
|  | 23c | Discuss any limitations of the review processes used. | Discussion, limitations |
|  | 23d | Discuss implications of the results for practice, policy, and future research. | Discussion  Conclusions |
| **OTHER INFORMATION** | | |  |
| Registration and protocol | 24a | Provide registration information for the review, including register name and registration number, or state that the review was not registered. | Abstract  Methods – Paragraph 1 |
|  | 24b | Indicate where the review protocol can be accessed, or state that a protocol was not prepared. | A protocol was not prepared |
|  | 24c | Describe and explain any amendments to information provided at registration or in the protocol. | Abstract  Methods – Paragraph 1 |
| Support | 25 | Describe sources of financial or non-financial support for the review, and the role of the funders or sponsors in the review. | Ackowledgments |
| Competing interests | 26 | Declare any competing interests of review authors. | Conflicts of Interest |
| Availability of data, code and other materials | 27 | Report which of the following are publicly available and where they can be found: template data collection forms; data extracted from included studies; data used for all analyses; analytic code; any other materials used in the review. | Data Availability |
