# Supplementary material for: Counseling Supporting HIV Self-Testing and Linkage to Care Among Men Who Have Sex With Men: Systematic Review and Meta-Analysis
Source: JMIR Public Health Surveill. 2024 Jan 24;10:e45647. doi: 10.2196/45647 (PMC10851126; doi:10.2196/45647)
Supplement: Multimedia Appendix 2 [file publichealth_v10i1e45647_app2.docx]

# Multimedia Appendix 2. Search terms

**MEDLINE**

| **Steps** | **Search terms** | **Results**  **06/30/2022** |
| --- | --- | --- |
| #1 | “HIV*”, OR “Human Immunodeficiency Virus*” | 371,555 |
| #2 | “self-test*”, OR “home-based test*”, OR “home test*” | 2637 |
| #3 | “counse*”, OR “interven*”, OR “support*”, OR “strateg*”, OR “approach*”, OR “innovat*”, OR “initiat*”, OR “implement*” | 12,767,826 |
| #4 | “social media” OR “mobile applica*” OR “app*” OR “mobile health” OR “Mhealth” OR “digital*” OR “online” OR “online real-time counsel*” OR “text messag*” OR “SMS*” OR “digital vending machin*” OR “community-led deliver*” OR “door-to-door distribut*” OR “secondary distribut*” OR “opinion leader*’ OR “community-based organi*” | 6,587,837 |
| #5 | “linkage to car*”, OR “linkage to service*”, OR “linkage to treat*”, OR “antiretroviral therap*”, OR “ART”, OR “PrEP”, OR “pre-exposure prophylaxis”, OR “Post-Exposure Prophylaxis”, OR “PEP”, OR “HIV test confirm*”, OR “blood sample confirm*” | 162,305 |
| #6 | “MSM”, OR “men who have sex with men”, OR “gay”, OR “bisexual men”, OR “sexual minority men”, OR “homosexual male*”. | 26,130 |
| #7 | #1 AND #2 | 1,011 |
| #8 | #7 AND 3 | 832 |
| #9 | #7 AND 4 | 594 |
| #10 | #8 OR #9 | 896 |
| #11 | #10 AND #5 AND #6 | 247 |

**Global Health**

| **Steps** | **Search terms** | **Results**  **06/30/2022** |
| --- | --- | --- |
| #1 | “HIV*”, OR “Human Immunodeficiency Virus*” | 209,135 |
| #2 | “self-test*”, OR “home-based test*”, OR “home test*” | 1,079 |
| #3 | “counse*”, OR “interven*”, OR “support*”, OR “strateg*”, OR “approach*”, OR “innovat*”, OR “initiat*”, OR “implement*” | 944,955 |
| #4 | “social media” OR “mobile applica*” OR “app*” OR “mobile health” OR “Mhealth” OR “digital*” OR “online” OR “online real-time counsel*” OR “text messag*” OR “SMS*” OR “digital vending machin*” OR “community-led deliver*” OR “door-to-door distribut*” OR “secondary distribut*” OR “opinion leader*’ OR “community-based organi*” | 1,040,060 |
| #5 | “linkage to car*”, OR “linkage to service*”, OR “linkage to treat*”, OR “antiretroviral therap*”, OR “ART”, OR “PrEP”, OR “pre-exposure prophylaxis”, OR “Post-Exposure Prophylaxis”, OR “PEP”, OR “HIV test confirm*”, OR “blood sample confirm*” | 62,276 |
| #6 | “MSM”, OR “men who have sex with men”, OR “gay”, OR “bisexual men”, OR “sexual minority men”, OR “homosexual male*”. | 30,561 |
| #7 | #1 AND #2 | 246 |
| #8 | #7 AND 3 | 170 |
| #9 | #7 AND 4 | 130 |
| #10 | #8 OR #9 | 205 |
| #11 | #10 AND #5 AND #6 | 152 |

**Web of Science**

| **Steps** | **Search terms** | **Results**  **06/30/2022** |
| --- | --- | --- |
| #1 | “HIV*”, OR “Human Immunodeficiency Virus*” | 475,639 |
| #2 | “self-test*”, OR “home-based test*”, OR “home test*” | 76,581 |
| #3 | “counse*”, OR “interven*”, OR “support*”, OR “strateg*”, OR “approach*”, OR “innovat*”, OR “initiat*”, OR “implement*” | 23,359,642 |
| #4 | “social media” OR “mobile applica*” OR “app*” OR “mobile health” OR “Mhealth” OR “digital*” OR “online” OR “online real-time counsel*” OR “text messag*” OR “SMS*” OR “digital vending machin*” OR “community-led deliver*” OR “door-to-door distribut*” OR “secondary distribut*” OR “opinion leader*’ OR “community-based organi*” | 21,549,462 |
| #5 | “linkage to car*”, OR “linkage to service*”, OR “linkage to treat*”, OR “antiretroviral therap*”, OR “ART”, OR “PrEP”, OR “pre-exposure prophylaxis”, OR “Post-Exposure Prophylaxis”, OR “PEP”, OR “HIV test confirm*”, OR “blood sample confirm*” | 13,578 |
| #6 | “MSM”, OR “men who have sex with men”, OR “gay”, OR “bisexual men”, OR “sexual minority men”, OR “homosexual male*”. | 120,319 |
| #7 | #1 AND #2 | 18,987 |
| #8 | #7 AND 3 | 1,896 |
| #9 | #7 AND 4 | 1,259 |
| #10 | #8 OR #9 | 2,053 |
| #11 | #10 AND #5 AND #6 | 421 |

**Embase**

| **Steps** | **Search terms** | **Results**  **06/30/2022** |
| --- | --- | --- |
| #1 | “HIV*”, OR “Human Immunodeficiency Virus*” | 555,277 |
| #2 | “self-test*”, OR “home-based test*”, OR “home test*” | 4,406 |
| #3 | “counse*”, OR “interven*”, OR “support*”, OR “strateg*”, OR “approach*”, OR “innovat*”, OR “initiat*”, OR “implement*” | 8,454,419 |
| #4 | “social media” OR “mobile applica*” OR “app*” OR “mobile health” OR “Mhealth” OR “digital*” OR “online” OR “online real-time counsel*” OR “text messag*” OR “SMS*” OR “digital vending machin*” OR “community-led deliver*” OR “door-to-door distribut*” OR “secondary distribut*” OR “opinion leader*’ OR “community-based organi*” | 9,829,977 |
| #5 | “linkage to car*”, OR “linkage to service*”, OR “linkage to treat*”, OR “antiretroviral therap*”, OR “ART”, OR “PrEP”, OR “pre-exposure prophylaxis”, OR “Post-Exposure Prophylaxis”, OR “PEP”, OR “HIV test confirm*”, OR “blood sample confirm*” | 10,489 |
| #6 | “MSM”, OR “men who have sex with men”, OR “gay”, OR “bisexual men”, OR “sexual minority men”, OR “homosexual male*”. | 41,231 |
| #7 | #1 AND #2 | 668 |
| #8 | #7 AND 3 | 521 |
| #9 | #7 AND 4 | 408 |
| #10 | #8 OR #9 | 584 |
| #11 | #10 AND #5 AND #6 | 419 |

**APA Psyclnfo**

| **Steps** | **Search terms** | **Results**  **06/30/2022** |
| --- | --- | --- |
| #1 | “HIV*”, OR “Human Immunodeficiency Virus*” | 60,088 |
| #2 | “self-test*”, OR “home-based test*”, OR “home test*” | 903 |
| #3 | “counse*”, OR “interven*”, OR “support*”, OR “strateg*”, OR “approach*”, OR “innovat*”, OR “initiat*”, OR “implement*” | 1,927,975 |
| #4 | “social media” OR “mobile applica*” OR “app*” OR “mobile health” OR “Mhealth” OR “digital*” OR “online” OR “online real-time counsel*” OR “text messag*” OR “SMS*” OR “digital vending machin*” OR “community-led deliver*” OR “door-to-door distribut*” OR “secondary distribut*” OR “opinion leader*’ OR “community-based organi*” | 1,723,371 |
| #5 | “linkage to car*”, OR “linkage to service*”, OR “linkage to treat*”, OR “antiretroviral therap*”, OR “ART”, OR “PrEP”, OR “pre-exposure prophylaxis”, OR “Post-Exposure Prophylaxis”, OR “PEP”, OR “HIV test confirm*”, OR “blood sample confirm*” | 62,276 |
| #6 | “MSM”, OR “men who have sex with men”, OR “gay”, OR “bisexual men”, OR “sexual minority men”, OR “homosexual male*”. | 30,561 |
| #7 | #1 AND #2 | 246 |
| #8 | #7 AND 3 | 170 |
| #9 | #7 AND 4 | 130 |
| #10 | #8 OR #9 | 205 |
| #11 | #10 AND #5 AND #6 | 78 |

**Scopus**

| **Steps** | **Search terms** | **Results**  **06/30/2022** |
| --- | --- | --- |
| #1 | “HIV*”, OR “Human Immunodeficiency Virus*” | 132,363 |
| #2 | “self-test*”, OR “home-based test*”, OR “home test*” | 13,260 |
| #3 | “counse*”, OR “interven*”, OR “support*”, OR “strateg*”, OR “approach*”, OR “innovat*”, OR “initiat*”, OR “implement*” | 20,016,375 |
| #4 | “social media” OR “mobile applica*” OR “app*” OR “mobile health” OR “Mhealth” OR “digital*” OR “online” OR “online real-time counsel*” OR “text messag*” OR “SMS*” OR “digital vending machin*” OR “community-led deliver*” OR “door-to-door distribut*” OR “secondary distribut*” OR “opinion leader*’ OR “community-based organi*” | 26,291,895 |
| #5 | “linkage to car*”, OR “linkage to service*”, OR “linkage to treat*”, OR “antiretroviral therap*”, OR “ART”, OR “PrEP”, OR “pre-exposure prophylaxis”, OR “Post-Exposure Prophylaxis”, OR “PEP”, OR “HIV test confirm*”, OR “blood sample confirm*” | 973,679 |
| #6 | “MSM”, OR “men who have sex with men”, OR “gay”, OR “bisexual men”, OR “sexual minority men”, OR “homosexual male*”. | 61,675 |
| #7 | #1 AND #2 | 518 |
| #8 | #7 AND 3 | 428 |
| #9 | #7 AND 4 | 320 |
| #10 | #8 OR #9 | 476 |
| #11 | #10 AND #5 AND #6 | 45 |
