# Supplementary material for: Counseling Supporting HIV Self-Testing and Linkage to Care Among Men Who Have Sex With Men: Systematic Review and Meta-Analysis
Source: JMIR Public Health Surveill. 2024 Jan 24;10:e45647. doi: 10.2196/45647 (PMC10851126; doi:10.2196/45647)
Supplement: Multimedia Appendix 3 [file publichealth_v10i1e45647_app3.docx]

**Multimedia Appendix 3. Studies were assessed using the National Institute of Health (NIH) quality assessment tool for controlled intervention studies.**

| **Study** | **Was the study described as randomized, a randomized trial, a randomized clinical trial, or an RCT?** | **Was the method of randomization adequate (i.e., use of randomly generated assignment)?** | **Was the treatment allocation concealed (so that assignments could not be predicted)?** | **Were study participants and providers blinded to treatment group assignment?** | **Were the people assessing the outcomes blinded to the participants' group assignments?** | **Were the groups similar at baseline on important characteristics that could affect outcomes (e.g., demographics, risk factors, co-morbid conditions)?** | **Was the overall drop-out rate from the study at endpoint 20% or lower of the number allocated to treatment?** | **Was the differential drop-out rate (between treatment groups) at endpoint 15 percentage points or lower?** | **Was there high adherence to the intervention protocols for each treatment group?** | **Were other interventions avoided or similar in the groups (e.g., similar background treatments)?** | **Were outcomes assessed using valid and reliable measures, implemented consistently across all study participants?** | **Did the authors report that the sample size was sufficiently large to be able to detect a difference in the main outcome between groups with at least 80% power?** | **Were outcomes reported or subgroups analyzed prespecified (i.e., identified before analyses were conducted)?** | **Were all randomized participants analyzed in the group to which they were originally assigned, i.e., did they use an intention-to-treat analysis?** | ***Overall quality (NIH%)** |
| --- | --- | --- | --- | --- | --- | --- | --- | --- | --- | --- | --- | --- | --- | --- | --- |
| Jamil 2017 [39] | Yes | Yes | Yes | Yes | Yes | Yes | Yes | Yes | Yes | Yes | Yes | Yes | Yes | NR | Good (93) |
| Katz 2018 [44] | Yes | Yes | Yes | Yes | Yes | Yes | Yes | Yes | Yes | Yes | Yes | No | Yes | No | Good (86) |
| Wray 2018 [48] | Yes | Yes | No | No | No | Yes | Yes | Yes | Yes | Yes | No | No | Yes | Yes | Fair (64) |
| Wang 2018 [49] | Yes | Yes | No | No | Yes | Yes | Yes | Yes | Yes | Yes | No | Yes | Yes | Yes | Good (79) |
| Zhu 2019 [56] | Yes | Yes | No | No | No | Yes | Yes | Yes | Yes | Yes | No | Yes | Yes | Yes | Fair (71) |
| Balán 2020 [57] | Yes | Yes | NR | NR | NR | Yes | Yes | Yes | Yes | Yes | Yes | No | NA | No | Fair (57) |
| Carballo-Diéguez 2020 [58] | Yes | Yes | NR | NR | NR | Yes | Yes | Yes | No | Yes | No | No | Yes | No | Fair (50) |
| MacGowan 2020 [61] | Yes | Yes | Yes | Yes | Yes | Yes | Yes | Yes | Yes | Yes | Yes | Yes | Yes | NR | Good (93) |
| Zhang 2020 [66] | Yes | Yes | Yes | Yes | Yes | Yes | Yes | Yes | Yes | Yes | No | Yes | Yes | NR | Good (86) |
| Cheng 2021 [70] | Yes | Yes | No | No | No | Yes | No | Yes | Yes | Yes | Yes | Yes | Yes | Yes | Fair (71) |
| Frye 2021 [79] | Yes | Yes | No | No | No | Yes | No | Yes | Yes | Yes | No | Yes | Yes | Yes | Fair (64) |
| Wirtz 2021 [83] | Yes | Yes | No | No | No | Yes | No | Yes | Yes | Yes | No | Yes | Yes | No | Fair (57) |

* Quality was poor (0–4 out of 14 questions), fair (5–10 out of 14 questions), good (11–14 out of 14 questions); NA: not applicable, NR: not reported; CD, cannot determine; NA, not applicable; NR, not reported. Criteria of ratings followed the statements listed in Quality Rating of National Heart Lung, and Blood Institute (NHLBI). Study Quality Assessment Tools 2022 [Available from: <https://www.nhlbi.nih.gov/health-topics/study-quality-assessment-tools>. The mean score from RCTs on the NIH Quality Assessment Scale was 73% (range from 50%-93%). No study was of poor quality; 7 studies had fair quality, and 5 had good quality.

**Multimedia Appendix 3. Studies were assessed using the National Institute of Health (NIH) quality assessment tool for observational cohort and cross-sectional studies.**

| **Study** | **Was the research question or objective in this paper clearly stated?** | **Was the study population clearly specified and defined?** | **Was the participation rate of eligible persons at least 50%?** | **Were all the subjects selected or recruited from the same or similar populations (including the same time period)? Were inclusion and exclusion criteria for being in the study prespecified and applied uniformly to all participants?** | **Was a sample size justification, power description, or variance and effect estimates provided?** | **For the analyses in this paper, were the exposure(s) of interest measured prior to the outcome(s) being measured?** | **Was the timeframe sufficient so that one could reasonably expect to see an association between exposure and outcome if it existed?** | **For exposures that can vary in amount or level, did the study examine different levels of the exposure as related to the outcome (e.g., categories of exposure, or exposure measured as continuous variable)?** | **Were the exposure measures (independent variables) clearly defined, valid, reliable, and implemented consistently across all study participants?** | **Was the exposure(s) assessed more than once over time?** | **Were the outcome measures (dependent variables) clearly defined, valid, reliable, and implemented consistently across all study participants?** | **Were the outcome assessors blinded to the exposure status of participants?** | **Was loss to follow-up after baseline 20% or less?** | **Were key potential confounding variables measured and adjusted statistically for their impact on the relationship between exposure(s) and outcome(s)?** | ***Overall quality (NIH%)** |
| --- | --- | --- | --- | --- | --- | --- | --- | --- | --- | --- | --- | --- | --- | --- | --- |
| Marlin 2014 [33] | Yes | Yes | Yes | Yes | No | No | No | Yes | No | No | No | No | No | No | Fair (36) |
| Tao 2014 [34] | Yes | Yes | NR | Yes | No | No | No | Yes | No | No | No | No | No | Yes | Fair (36) |
| Huang 2016 [36] | Yes | Yes | Yes | Yes | No | No | No | Yes | No | No | No | No | Yes | No | Fair (43) |
| Rosengren 2016 [37] | Yes | Yes | NR | Yes | No | No | Yes | Yes | No | No | No | No | No | No | Fair (36) |
| Volk 2016 [38] | Yes | Yes | Yes | Yes | No | Yes | Yes | Yes | No | No | No | No | Yes | No | Fair (57) |
| Qin 2017 [40] | Yes | Yes | Yes | Yes | No | No | No | Yes | Yes | No | Yes | No | No | Yes | Fair (57) |
| Zhong 2017 [41] | Yes | Yes | Yes | Yes | No | No | No | Yes | No | No | No | No | No | Yes | Fair (43) |
| Choko 2018 [42] | Yes | Yes | Yes | Yes | No | Yes | Yes | Yes | No | Yes | No | No | Yes | No | Fair (64) |
| Green 2018 [43] | Yes | Yes | NR | Yes | No | No | No | Yes | No | No | No | No | No | Yes | Fair (36) |
| Lippman 2018 [45] | Yes | Yes | Yes | Yes | No | Yes | Yes | Yes | No | No | No | No | Yes | No | Fair (57) |
| Pant Pai 2018 [46] | Yes | Yes | Yes | Yes | Yes | No | No | Yes | Yes | No | No | No | No | No | Fair (50) |
| Tun 2018 [47] | Yes | Yes | Yes | Yes | No | Yes | Yes | Yes | No | Yes | No | No | Yes | No | Fair (64) |
| Jin 2019 [50] | Yes | Yes | Yes | Yes | No | No | No | Yes | No | No | Yes | No | No | Yes | Fair (50) |
| De Boni 2019 [51] | Yes | Yes | Yes | Yes | No | No | No | Yes | Yes | No | No | No | No | NR | Fair (43) |
| Hidayat 2019 [27] | Yes | Yes | Yes | Yes | No | No | No | Yes | No | No | Yes | No | No | No | Fair (43) |
| Nguyen 2019 [53] | Yes | Yes | Yes | Yes | No | No | No | Yes | No | No | No | No | No | No | Fair (36) |
| Vera (2019) [54] | Yes | Yes | NR | Yes | No | No | No | Yes | No | No | Yes | No | No | NR | Fair (36) |
| Wesolowski 2019 [55] | Yes | Yes | Yes | Yes | No | Yes | Yes | Yes | No | No | No | No | No | No | Fair (50) |
| Edelstein 2020 [59] | Yes | Yes | Yes | Yes | No | Yes | Yes | Yes | No | Yes | No | No | No | Yes | Fair (64) |
| Johnson 2020 [60] | Yes | Yes | Yes | Yes | No | Yes | Yes | Yes | No | Yes | No | No | No | NA | Fair (57) |
| Okoboi 2020 [62] | Yes | Yes | NR | Yes | No | No | No | Yes | Yes | No | Yes | No | No | No | Fair (43) |
| Phanuphak 2020 [63] | Yes | Yes | Yes | Yes | Yes | Yes | Yes | Yes | No | No | No | No | No | Yes | Fair (64) |
| Yan 2020 [64] | Yes | Yes | Yes | Yes | No | Yes | Yes | Yes | No | No | Yes | No | Yes | Yes | Fair (71) |
| Wang 2020 [65] | Yes | Yes | Yes | Yes | No | Yes | Yes | Yes | Yes | Yes | Yes | No | No | Yes | Good (79) |
| Zhang 2020 [67] | Yes | Yes | Yes | Yes | No | No | No | Yes | No | No | Yes | No | No | Yes | Fair (50) |
| Bell 2021 [68] | Yes | Yes | Yes | Yes | No | No | No | Yes | Yes | No | No | No | No | Yes | Fair (50) |
| Chen (2021) [69] | Yes | Yes | NR | Yes | No | Yes | Yes | Yes | Yes | Yes | No | No | Yes | No | Fair (64) |
| Chan 2021 [71] | Yes | Yes | Yes | Yes | No | Yes | Yes | Yes | Yes | Yes | Yes | No | Yes | Yes | Good (86) |
| Hecht 2021 [72] | Yes | Yes | NR | Yes | No | No | No | Yes | Yes | No | No | No | No | No | Fair (36) |
| Li 2021 [73] | Yes | Yes | Yes | Yes | Yes | No | No | Yes | No | No | No | No | No | Yes | Fair (50) |
| da Cruz 2021 [74] | Yes | Yes | NR | Yes | No | No | No | Yes | Yes | No | No | No | No | NA | Fair (36) |
| Wu 2021 [75] | Yes | Yes | Yes | Yes | No | Yes | Yes | Yes | No | Yes | Yes | No | Yes | NA | Fair (71) |
| Zhang 2021 [76] | Yes | Yes | Yes | Yes | No | Yes | Yes | Yes | Yes | No | No | No | Yes | Yes | Fair (71) |
| Abubakari 2021 [77] | Yes | Yes | Yes | Yes | No | No | No | No | Yes | No | No | No | No | No | Fair (36) |
| Maatouk 2021 [78] | Yes | Yes | Yes | Yes | No | No | No | Yes | No | No | No | No | No | No | Fair (36) |
| Girault 2021 [80] | Yes | Yes | Yes | Yes | No | No | No | Yes | No | No | No | No | No | No | Fair (36) |
| Phongphiew 2021 [81] | Yes | Yes | NR | Yes | Yes | No | No | Yes | No | No | Yes | No | No | No | Fair (43) |
| Widyanthini 2021 [82] | Yes | Yes | NR | Yes | No | No | Yes | Yes | No | No | No | No | No | No | Fair (36) |
| Dijkstra 2021 [84] | Yes | Yes | Yes | Yes | No | No | No | Yes | No | No | Yes | No | No | No | Fair (43) |
| O’Byrne 2021 [85] | Yes | Yes | Yes | Yes | No | No | No | Yes | No | No | Yes | No | No | No | Fair (43) |
| Lillie 2021 [86] | Yes | Yes | NR | Yes | No | Yes | Yes | Yes | No | No | No | No | No | Yes | Fair (50) |

* Quality was poor (0–4 out of 14 questions), fair (5–10 out of 14 questions), good (11–14 out of 14 questions); NA: not applicable, NR: not reported; CD, cannot determine; NA, not applicable; NR, not reported. Criteria of ratings followed the statements listed in the Quality Rating of the National Heart Lung and Blood Institute (NHLBI). Study Quality Assessment Tools 2022 [Available from: <https://www.nhlbi.nih.gov/health-topics/study-quality-assessment-tools>. The mean score from observational studies was 50% (range from 36%-86%). Two conference abstracts were evaluated with this scale, but a final score could not be calculated due to a lack of detailed methods and reporting of outcome metrics. No study was of poor quality; 39 studies had fair quality, and 2 had good quality
