# Supplementary material for: Counseling Supporting HIV Self-Testing and Linkage to Care Among Men Who Have Sex With Men: Systematic Review and Meta-Analysis
Source: JMIR Public Health Surveill. 2024 Jan 24;10:e45647. doi: 10.2196/45647 (PMC10851126; doi:10.2196/45647)
Supplement: Multimedia Appendix 4 [file publichealth_v10i1e45647_app4.docx]

**Multimedia Appendix 4. Summary of active counseling support in included studies**

| **Study (first author) and country** | **Pre-test counseling (essential components)** | | | | | **Pre-test counseling (other components)** | | | | | **Post-test counseling (essential components)** | | | |
| --- | --- | --- | --- | --- | --- | --- | --- | --- | --- | --- | --- | --- | --- | --- |
|  | **Informing the participants of the benefit of taking the test and the implications of both negative and positive results** | **Assessing the risks and window periods** | **Assuring client’s right to refuse to take the test** | **Encouraging the client to anticipate the possibility of beneficial disclosure of sero-status** | **Providing preventive information and materials** | **Asking reason for HIV testing** | **Sharing stories about general health concerns**  **of MSM** | **Assessing potential social support** | **Introducing first-person stories about people**  **diagnosed and living with HIV** | **Providing local data, news, and policies about HIV and**  **STI infections among MSM** | **Interpreting testing results** | **Providing HIV-negative individuals with prevention information and material** | **Providing HIV-positive individuals with psychological support, beneficial disclosure of positive sero-status, and referral for further care, treatment, and support services** | |
| Marlin (2014) [33] USA | - | - | - | - | - | - | - | - | - | - | Yes | - | Yes | |
| Tao (2014) [34] China | Yes | Yes | Yes | Yes | Yes | - | - | - | - | - | Yes | Yes | Yes | |
| Sabharwal (2015) [35] USA | - | - | - | - | - | - | - | - | - | - | - | - | Yes | |
| Huang (2016) [36] USA | - | - | - | - | - | - | - | - | - | - | Yes | - | Yes | |
| Rosengren (2016) [37] USA | - | - | - | - | - | - | - | - | - | - | Yes | - | Yes | |
| Volk (2016) [38] Brazil | - | - | - |  | Yes | - | - | - | - | Yes | Yes | Yes | Yes | |
| Jamil (2017) [39] Australia | - | - | - |  |  | - | - | - | - | - | Yes | - | Yes | |
| Qin (2017) [40] China | - | - | - |  |  | - | - | - | - | - | Yes | - | Yes | |
| Zhong (2017) [41]  China | - | - | - |  |  | - | - | - | - | - | Yes | - | Yes | |
| Choko (2018) [42] Uganda | - | - | - |  |  | - | - | - | - | - | Yes | - | Yes | |
| Green (2018) [43] Vietnam | - | - | - |  |  | - | - | - | - | - | Yes | - | Yes | |
| Katz (2018) [44] USA | Yes | Yes | Yes | Yes | Yes | - | - | - | - | Yes | Yes | Yes | Yes | |
| Lippman (2018) [45] South Africa | - | - | - |  |  | - | - | - | - | Yes | Yes | Yes | Yes | |
| Pant Pai (2018) [46] Canada | Yes | Yes | Yes | Yes | Yes | - | - | - | - | - | Yes | Yes | Yes | |
| Tun (2018) [47] Nigeria, West Africa | - | - | - | - | Yes | - | - | - | - | - | Yes | - | Yes | |
| Wray (2018) [48] USA | Yes | Yes | Yes | Yes | Yes | - | - | - | - | Yes | Yes | - | Yes | |
| Wang (2018) [49] China | Yes | Yes | Yes | Yes | Yes | - | - | - | - | - | Yes | Yes | Yes | |
| De Boni (2019) [51] Brazil | Yes | Yes | - | - | Yes | - | - |  | - | Yes | - | - | Yes | |
| Gashobotse (2019) [52] Burundi, East Africa | - | - | - | - | - | - | - | - | - | - | - | - | Yes | |
| Nguyen (2019) [53] Vietnam | Yes | Yes | - | - | - | - | - | - | - | - | Yes | - | Yes | |
| Vera (2019) [54] UK | - | - | - |  |  | - | - | - | - | - | Yes | - | Yes | |
| Wesolowski (2019) [55] USA | Yes | - | - |  |  | - | - | - | - | - | Yes | - | Yes | |
| Zhu (2019) [56] China | Yes | - | - | - | Yes | - | Yes | - | Yes | Yes | Yes | Yes | Yes | |
| Balán (2020) [57] USA | - | - | Yes | - | - | - | - | Yes | - | - | - | - | Yes | |
| Carballo-Diéguez (2020) [58] USA | - | - | Yes | - | - | - | - | Yes | - | - | - | - | Yes | |
| Johnson (2020) [60] USA | - | - | - | - | Yes | - | - | - | - | Yes | - | Yes | Yes | |
| Okoboi (2020) [62] Uganda | Yes | - | - | - | - | - | - | - | - | - | - | - | Yes | |
| Phanuphak (2020) [63] Thailand | Yes | Yes | Yes | Yes | Yes | - | - | - | - | - | Yes | Yes | Yes | |
| Yan (2020) [64] China | Yes | - | - | Yes | - | - | - | - | - | - | Yes | - | Yes | |
| Wang (2020) [65] China | Yes | Yes | Yes | Yes | Yes | - | - | - | - | - | Yes | - | Yes | |
| Zhang (2020) [66] China | - | - | - | - | - | - | - | Yes | - | - | Yes | - | Yes | |
| Zhang (2020) [67] China | Yes | Yes | Yes | Yes | Yes | - | - | - | - | - | Yes | - | Yes | |
| Bell (2021) [68] Australia | Yes | Yes | Yes | Yes | Yes | Yes | - | - | - | - | Yes | Yes | Yes | |
| Chen (2021) [69] South Africa | Yes | - | - | Yes | Yes | - | - | - | - | - | Yes | - | Yes | |
| Cheng (2021) [70] China | Yes | - | Yes | - | Yes | - | - | - | - | - | Yes | Yes | Yes | |
| Chan (2021) [71] China | Yes | Yes | Yes | Yes | Yes | - | - | - | - | - | Yes | Yes | Yes | |
| Hecht (2021) [72] USA | - | - | - | - | - | - | - | - | - | - | Yes | Yes | Yes | |
| Li (2021) [73] China | - | - | - | - | - | - | - | - | - | - | Yes | - | Yes | |
| da Cruz (2021) [74] Brazil | - | - | - | - | - | - | - | - | - | - | - | - | Yes | |
| Wu (2021) [75] China | - | - | - | - | - | - | - | - | - | - | - | - | Yes | |
| Zhang (2021) [76] China | Yes | - | Yes | - | - | - | - | - | - | - | Yes | Yes | Yes | |
| Abubakari (2021) [77] Ghana | - | - | - | - | - | - | - | - |  |  | Yes | Yes | Yes | |
| Maatouk (2021) [78] Lebanon | - | - | - | - | - | - | - | - | - | - | Yes | - | Yes | |
| Frye (2021) [79] USA | Yes | Yes | Yes | Yes | Yes | - | - | Yes | - | - | Yes | Yes | Yes | |
| Phongphiew (2021) [81] Thailand | Yes | Yes | Yes | Yes | Yes | - | - | - | - | - | Yes | Yes | Yes | |
| Widyanthini (2021) [82] Indonesia | Yes | Yes | Yes | - | - | - | - | - | - | - | Yes | - | Yes | |
| Wirtz (2021) [83] Myanmar | Yes | - | Yes | - | - | - | Yes | - | - | - | Yes | - | Yes | |
| Dijkstra (2021) [84] Kenya, East Africa | - | - | - | - | - | - | - | - | - | - | Yes | - | Yes | |
| O'Byrne (2021) [85] Canada | Yes | Yes | Yes | Yes | - | - | - | - | - | - | Yes | Yes | Yes | |
| Lillie (2021) [86] Burundi, East Africa | Yes | Yes | Yes | Yes | Yes | - | - | - | - | - | Yes | Yes | Yes | |
| Total | 25/50 | 17/50 | 20/50 | 16/50 | 20/50 | 1/50 | 2/50 | 4/50 | 1/50 | 7/50 | 41/50 | 19/50 | 50/50 | |
|  |  |  |  |  |  |  |  |  |  |  |  |  |  |  |

“-” Not applicable

**Multimedia Appendix 4. Summary of passive counseling support in included studies**

| **Study (first author) and country** | **Pre-test counseling (essential components)** | | | | | **Pre-test counseling (other components)** | | | | | **Post-test counseling (essential components)** | | |
| --- | --- | --- | --- | --- | --- | --- | --- | --- | --- | --- | --- | --- | --- |
|  | **Informing the participants of the benefit of taking the test and the implications of both negative and positive results** | **Assessing the risks and window periods** | **Assuring client’s right to refuse to take the test** | **Encouraging the client to anticipate the possibility of beneficial disclosure of sero-status** | **Providing preventive information and materials** | **Asking reason for HIV testing** | **Sharing stories about general health concerns**  **of MSM** | **Assessing potential social support** | **Introducing first-person stories about people**  **diagnosed and living with HIV** | **Providing local data, news, and policies about HIV and**  **STI infections among MSM** | **Interpreting testing results** | **Providing HIV-negative individuals with prevention information and material** | **Providing HIV-positive individuals with psychological support, beneficial disclosure of positive sero-status, and referral for further care, treatment, and support services** |
| Jin (2019) [50] China | - | - | - |  |  | - | - | - | - | - | - | - | Yes, only if they were interested in linkage services. |
| Hidayat (2019) [27] Indonesia | - | - | - |  |  | - | - | - | - |  | Yes, only determining how many lines appear on the result display. | - | Yes, only determining how many lines appear on the result display. |
| Edelstein (2020) [59] USA | Yes | - | - | - | Yes | - | - | - | - |  | - | Yes, when there is a need | Yes, when there is a need. |
| MacGowan (2020) [61] USA | Yes | - | - |  | Yes | - | - | - | - | Yes | - | - | Yes, when requested |
| Girault (2021) [80] Thailand | Yes | - | - | - | - | - | - | - | - | - | Yes, when requested | - | - |

“-” Not applicable
