# Supplementary material for: Counseling Supporting HIV Self-Testing and Linkage to Care Among Men Who Have Sex With Men: Systematic Review and Meta-Analysis
Source: JMIR Public Health Surveill. 2024 Jan 24;10:e45647. doi: 10.2196/45647 (PMC10851126; doi:10.2196/45647)
Supplement: Multimedia Appendix 5 [file publichealth_v10i1e45647_app5.docx]

**Multimedia Appendix 5. Summary of Sensitivity Analysis**

| **Linkage to care** | **Active counseling support** | | | **Passive counseling support** | | |
| --- | --- | --- | --- | --- | --- | --- |
|  | **Pooled proportion, % (95% CI)** | ***I*^2^** | **The study was removed** | **Range of pooled proportion, % (95% CI)** | ***I*^2^** | **The study was removed** |
| Reporting test results | 98.3% (95% CI=85.6-99.8%) | 96.5% | De Boni et al., 2019 [51] | - | - | - |
|  | 97.4% (95% CI=65.4-99.9%) | 99.7% | Zhong et al., 2017 [41] | - | - | - |
|  | 97.1% (95% CI=64.4-99.8%) | 99.7% | Zhu et al., 2019 [56] | - | - | - |
|  | 94.1% (95% CI=67.9-99.2%) | 99.7% | Yan et al., 2020 [64] | - | - | - |
|  | 95.3% (95% CI=65.9-99.5%) | 99.7% | Zhang et al., 2020 [66] | - | - | - |
|  | 97.8% (95% CI=69.9-99.9%) | 99.6% | Zhang et al., 2020 [67] | - | - | - |
|  | 98.0% (95% CI=72.9-99.9%) | 99.6% | Li et al., 2021 [73] | - | - | - |
|  | 96.8% (95% CI=62.5-99.8%) | 99.7% | Wu et al., 2021 [75] | - | - | - |
|  | 98.0% (95% CI=74.2-99.9%) | 99.7% | Zhang et al., 2021 [76] | - | - | - |
| Laboratory confirmation | 92.5% (95% CI=85.9-96.1%) | 58.2% | Jamil et al., 2017 [39] | 86.0% (95% CI=9.4-99.7%) | 86.6% | MacGowan et al., 2020 [61] |
|  | 92.6% (95% CI=86.0-96.2%) | 58.2% | Bell et al., 2021 [68] | 80.6% (95% CI=4.5-99.7%) | 62.2% | Edelstein et al., 2020 [59] |
|  | 92.5% (95% CI=85.9-96.1%) | 58.2% | Pant Pai et al., 2018 [46] | 55.5% (95% CI=27.0-80.8%) | 86.6% | Jin et al., 2019 [50] |
|  | 92.5% (95% CI=86.0-96.2%) | 58.2% | Huang et al., 2016 [36] | 82.7% (95% CI=5.2-99.8%) | 81.2% | Girault et al., 2021 [80] |
|  | 92.5% (95% CI=86.0-96.2%) | 58.2% | Rosengren et al., 2016 [37] | 86.4% (95% CI=10.4-99.7%) | 72.8% | Hidayat et al., 2019 [27] |
|  | 93.0% (95% CI=86.3-96.6%) | 58.1% | Carballo-Diéguez et al, 2020 [58] | - | - | - |
|  | 92.9% (95% CI=86.1-96.6%) | 58.2% | Johnson et al., 2018 [60] | - | - | - |
|  | 92.4% (95% CI=85.8-96.1%) | 58.2% | Katz et al., 2018 [44] | - | - | - |
|  | 92.5% (95% CI=86.0-96.2%) | 58.2% | Frye et al., 2021 [79] | - | - | - |
|  | 93.2% (95% CI=86.1-96.8%) | 57.9% | De Boni et al., 2019 [51] | - | - | - |
|  | 93.3% (95% CI=86.4-96.9%) | 57.3% | da Cruz et al., 2021 [74] | - | - | - |
|  | 91.5% (95% CI=84.9-95.3%) | 58.2% | Tao et al., 2014 [34] | - | - | - |
| Laboratory confirmation | 92.2% (95% CI=85.7-95.9%) | 58.2% | Zhong et al., 2017 [41] | - | - | - |
|  | 92.6% (95% CI=86.0-96.2%) | 58.2% | Wang et al., 2018 [65] | - | - | - |
|  | 92.5% (95% CI=86.0-96.2%) | 58.2% | Zhu et al., 2019 [56] | - | - | - |
|  | 92.5% (95% CI=85.9-96.1%) | 58.2% | Zhang et al., 2020 [66] | - | - | - |
|  | 92.4% (95% CI=85.8-96.1%) | 58.2% | Chan et al., 2021 [71] | - | - | - |
|  | 93.1% (95% CI=87.0-96.4%) | 48.6% | Li et al., 2021 [73] | - | - | - |
|  | 93.3% (95% CI=86.5-96.8%) | 58.1% | Wu et al., 2021 [75] | - | - | - |
|  | 91.3% (95% CI=84.8-95.2%) | 58.2% | Lippman et al., 2018 [45] | - | - | - |
|  | 92.5% (95% CI=85.9-96.1%) | 58.2% | Chen et al., 2021 [69] | - | - | - |
|  | 93.4% (95% CI=86.6-96.8%) | 57.9% | Phanuphak et al., 2020 [63] | - | - | - |
|  | 93.3% (95% CI=86.7-96.8%) | 57.6% | Qin et al., 2017 [40] | - | - | - |
|  | 92.2% (95% CI=85.6-95.9%) | 58.2% | Wang et al., 2020 [65] | - | - | - |
|  | 92.5% (95% CI=86.0-96.2%) | 58.2% | Phongphiew et al., 2021 [81] | - | - | - |
| Laboratory confirmation | 92.5% (95% CI=86.0-96.2%) | 58.2% | Maatouk et al., 2021 [78] | - | - | - |
|  | 93.1% (95% CI=87.0-96.4%) | 35.2% | Widyanthini et al., 2021 [82] | - | - | - |
|  | 92.5% (95% CI=86.0-96.2%) | 58.2% | Wirtz et al., 2021 [83] | - | - | - |
|  | 92.0% (95% CI=85.4-95.8%) | 58.2% | Tun et al., 2018 [47] | - | - | - |
|  | 93.2% (95% CI=86.0-96.8%) | 50.5% | Green et al., 2018 [43] | - | - | - |
|  | 92.5% (95% CI=85.0-96.4%) | 41.0% | Nguyen et al., 2019 [53] | - | - | - |
|  | 93.4% (95% CI=86.7-96.8%) | 57.7% | Gashobotse et al., 2019 [52] | - | - | - |
|  | 92.6% (95% CI=85.3-96.4%) | 55.3% | Lillie et al., 2021 [86] | - | - | - |
|  | 92.4% (95% CI=85.8-96.1%) | 58.2% | Choko et al., 2018 [42] | - | - | - |
|  | 92.2% (95% CI=85.7-95.9%) | 58.2% | Okoboi et al., 2020 [62] | - | - | - |
| Antiretroviral Therapy (ART) initiation | 90.7% (95% CI=86.7-93.6%) | 0% | Rosengren et al., 2016 [37] | 80.5% (95% CI=17.0-98.8%) | 0% | Edelstein et al., 2020 [59] |
|  | 90.6% (95% CI=86.6-93.5%) | 0% | Johnson et al., 2020 [60] | 85.2% (95% CI=23.9-99.1%) | 0% | MacGowan et al., 2020 [61] |
|  | 90.7% (95% CI=86.7-93.7%) | 0% | Katz et al., 2018 [44] | 85.7% (95% CI=26.8-99.0%) | 0% | Jin et al., 2019 [50] |
|  | 90.7% (95% CI=86.7-93.7%) | 0% | Wang et al., 2018 [49] | 74.1% (95% CI=56.7-86.2%) | 0% | Girault et al., 2021 [80] |
|  | 90.7% (95% CI=86.6-93.6%) | 0% | Zhang et al., 2020 [66] | - | - | - |
|  | 90.5% (95% CI=86.3-93.6%) | 0% | Zhang et al., 2020 [67] | - | - | - |
|  | 90.7% (95% CI=86.6-93.6%) | 0% | Chan et al., 2021 [71] | - | - | - |
|  | 90.7% (95% CI=86.6-93.6%) | 0% | Cheng et al., 2021 [70] | - | - | - |
|  | 90.2% (95% CI=86.2-93.1%) | 0% | Li et al., 2021 [73] | - | - | - |
|  | 90.9% (95% CI=86.7-93.9%) | 0% | Lippman et al., 2018 [45] | - | - | - |
|  | 91.4% (95% CI=89.1-93.3%) | 0% | Phanuphak et al., 2020 [63] | - | - | - |
|  | 90.7% (95% CI=86.7-93.6%) | 0% | Phongphiew et al., 2021 [81] | - | - | - |
|  | 90.7% (95% CI=86.7-93.6%) | 0% | Maatouk et al., 2021 [78] | - | - | - |
|  | 90.9% (95% CI=86.3-94.0%) | 0% | Widyanthini et al., 2021 [82] | - | - | - |
|  | 91.0% (95% CI=86.6-94.0%) | 0% | Dijkstra et al., 2021 [84] | - | - | - |
|  | 90.4% (95% CI=86.4-93.3%) | 0% | Tun et al., 2018 [47] | - | - | - |
|  | 91.2% (95% CI=86.1-94.6%) | 0% | Green et al., 2018 [43] | - | - | - |
|  | 90.2% (95% CI=85.0-93.8%) | 0% | Nguyen et al., 2019 [53] | - | - | - |
|  | 91.4% (95% CI=86.9-94.4%) | 0% | Gashobotse et al., 2019 [52] | - | - | - |
|  | 91.3% (95% CI=86.9-94.4%) | 0% | Lillie et al., 2021 [86] | - | - | - |
|  | 90.5% (95% CI=86.5-93.5%) | 0% | Okoboi et al., 2020 [62] | - | - | - |
| Referral to physicians | 96.2% (95% CI=84.6-98.1%) | 0% | Jamil et al., 2017 [39] | - | - | - |
|  | 96.3% (95% CI=84.8-99.2%) | 0% | Bell et al., 2021 [68] | - | - | - |
|  | 96.2% (95% CI=84.6-99.1%) | 0% | Pant Pai et al., 2018 [46] | - | - | - |
|  | 96.3% (95% CI=84.8-99.2%) | 0% | Vera et al., 2019 [54] | - | - | - |
|  | 96.2% (95% CI=84.6-99.1%) | 0% | Marlin et al., 2014 [33] | - | - | - |
|  | 96.2% (95% CI=84.7-99.2%) | 0% | Huang et al., 2016 [36] | - | - | - |
|  | 96.2% (95% CI=84.7-99.2%) | 0% | Wesolowski et al., 2019 [55] | - | - | - |
| Referral to physicians | 95.1% (95% CI=82.9-98.7%) | 0% | Balán et al., 2020 [57] | - | - | - |
|  | 97.3% (95% CI=83.7-99.6%) | 0% | Johnson et al., 2020 [60] | - | - | - |
|  | 96.7% (95% CI=85.2-99.3%) | 0% | Sabharwal et al., 2015 [35] | - | - | - |
|  | 96.1% (95% CI=84.5-99.1%) | 0% | Katz et al., 2018 [44] | - | - | - |
|  | 96.2% (95% CI=84.7-99.2%) | 0% | Frye et al., 2020 [79] | - | - | - |
|  | 96.2% (95% CI=84.7-99.2%) | 0% | Volk et al., 2016 [38] | - | - | - |
|  | 97.6% (95% CI=85.3-99.7%) | 0% | da Cruz et al., 2021 [74] | - | - | - |
|  | 95.9% (95% CI=84.2-99.0%) | 0% | Zhong et al., 2017 [41] | - | - | - |
|  | 96.3% (95% CI=84.8-99.2%) | 0% | Wang et al., 2018 [49] | - | - | - |
|  | 96.2% (95% CI=84.7-99.2%) | 0% | Zhu et al., 2019 [56] | - | - | - |
|  | 96.2% (95% CI=84.6-99.1%) | 0% | Zhang et al., 2020 [66] | - | - | - |
|  | 96.1% (95% CI=84.5-99.1%) | 0% | Cheng et al., 2021 [70] | - | - | - |
|  | 96.1% (95% CI=84.5-99.1%) | 0% | Chan et al., 2021 [71] | - | - | - |
| Referral to physicians | 97.5% (95% CI=85.3-99.6%) | 0% | Wu et al., 2021 [75] | - | - | - |
|  | 94.3% (95% CI=81.6-98.4%) | 0% | Lippman et al., 2018 [45] | - | - | - |
|  | 96.2% (95% CI=84.6-99.1%) | 0% | Chen et al., 2021 [69] | - | - | - |
|  | 97.5% (95% CI=85.6-99.6%) | 0% | Phanuphak et al., 2020 [63] | - | - | - |
|  | 95.8% (95% CI=84.1-99.0%) | 0% | Wang et al., 2020 [65] | - | - | - |
|  | 96.2% (95% CI=84.7-99.2%) | 0% | Phongphiew et al., 2021 [81] | - | - | - |
|  | 97.6% (95% CI=84.7-99.7%) | 0% | Abubakari et al., 2021 [77] | - | - | - |
|  | 95.5% (95% CI=83.7-98.9%) | 0% | Tun et al., 2018 [47] | - | - | - |
| Prevention strategies | 100.0% (95% CI=0-100.0%) | 0% | Tao et al., 2014 [34] | - | - | - |
|  | 100.0% (95% CI=0-100.0%) | 0% | Wang et al., 2018 [49] | - | - | - |
|  | 100.0% (95% CI=0-100.0%) | 0% | Johnson et al., 2020 [60] | - | - | - |
|  | 100.0% (95% CI=0-100.0%) | 0% | Phanuphak et al., 2020 [63] | - | - | - |
|  | 100.0% (95% CI=0-100.0%) | 0% | Chan et al, 2021 [71] | - | - | - |
|  | 100.0% (95% CI=0-100.0%) | 0% | Phongphiew et al., 2021 [81] | - | - | - |
|  | 100.0% (95% CI=0-100.0%) | 0% | O'Byrne et al., 2021 [85] | - | - | - |
| Pre-exposure prophylaxis (PrEP) initiation | 25.2% (95% CI=7.1-59.9%) | 97.8% | Wray et al., 2018 [48] | - | - | - |
|  | 23.6% (95% CI=6.8-56.7%) | 94.9% | Johnson et al., 2020 [60] | - | - | - |
|  | 33.8% (95% CI=13.4-62.9%) | 94.9% | Hecht et al., 2021 [72] | - | - | - |
|  | 21.0% (95% CI=7.6-46.3%) | 95.4% | O'Byrne et al., 2021 [85] | - | - | - |
|  | 32.0% (95% CI=11.3-63.6%) | 97.8% | Phongphiew et al., 2021 [81] | - | - | - |
|  | 27.6% (95% CI=7.5-64.1%) | 97.9% | Dijkstra et al., 2021 [84] | - | - | - |

“-”: not applicable
