# Supplementary material for: Counseling Supporting HIV Self-Testing and Linkage to Care Among Men Who Have Sex With Men: Systematic Review and Meta-Analysis
Source: JMIR Public Health Surveill. 2024 Jan 24;10:e45647. doi: 10.2196/45647 (PMC10851126; doi:10.2196/45647)
Supplement: Multimedia Appendix 6 [file publichealth_v10i1e45647_app6.docx]

**Multimedia Appendix 6. Univariate and Multivariable Metaregression Analyses of Linkage to care by Study Characteristics**

| **Stratified variables** | **Univariate** | | **Multivariate** | |
| --- | --- | --- | --- | --- |
|  | **Coefficient** | **P value** | **Coefficient** | **P value** |
| 1. ***Linkage to care among all included studies*** | | | | |
| Passive counseling (vs. active counseling) | -1.38 | **.04** | -0.63 | .47 |
| Year of the study in 2017 and later (vs. 2016 and earlier) | -1.42 | .27 | -1.96 | .19 |
| Sample size with more than or equal 300 (vs. less than 300) | -1.33 | **.02** | -1.41 | **.03** |
| Low-income countries (vs. lower-middle income-, upper-middle-income- and high-income-countries) | 0.15 | .57 | 0.61 | .14 |
| Peer and community counseling delivering modes (vs. technology and mobile health) | -0.50 | .39 | -1.33 | .**05** |
| Quality of counseling (the number of essential components) | 0.22 | **.05** | 0.24 | **.04** |
| 1. ***Linkage to care among studies that were provided active counseling along with HIVST*** | | | | |
| Year of the study in 2017 and later (vs. 2016 and earlier) | -2.01 | .17 | -2.22 | .16 |
| Sample size with more than or equal 300 (vs. less than 300) | -1.39 | **.04** | -1.47 | **.03** |
| Low-income countries (vs. lower-middle income-, upper-middle-income- and high-income-countries) | -.14 | .71 | .63 | .15 |
| Peer and community counseling delivering modes (vs. technology and mobile health) | -0.66 | .30 | -1.45 | **.05** |
| Both pre-test and post-test counseling (vs. post-test counseling) | 1.02 | **.09** | 0.75 | .34 |
| Quality of counseling (the number of essential components) | 0.22 | **.07** | 0.18 | .24 |
| 1. ***Linkage to care among studies that were provided passive counseling along with HIVST (Multivariate model not performed due to less than two significant factors)*** | | | | |
| Year of the study in 2017 and later (vs. 2016 and earlier) | - | - | - | - |
| Sample size with more than or equal 300 (vs. less than 300) | - | **-** | - | **-** |
| Low-income countries (vs. lower-middle income-, upper-middle-income- and high-income-countries) | 0.10 | .91 | - | - |
| Peer and community counseling delivering modes (vs. technology and mobile health) | -0.27 | .83 | - | - |
| Quality of counseling (the number of essential components) | -.51 | .29 | - | - |

**Multimedia Appendix 6.** **Sub-group Meta-analysis and Metaregression Analyses of Linkage to Care by Study Characteristics**

| **Stratified variables** | **Number of estimates** | **Pooled estimate, % (95% CI)** | | ***I*^2^** | **Univariate** | | | | | **Multivariate^1^** | | | |
| --- | --- | --- | --- | --- | --- | --- | --- | --- | --- | --- | --- | --- | --- |
|  |  |  |  |  | **Coefficient** | | **P value** | | | **Coefficient** | **P value** | |  |
| ***Linkage to reporting testing results*** | | | | | | | | |  |  |  |  |  |
| Year of the study |  |  | |  | - | | - | | | - | - | |  |
| 2016 and earlier | 0 | - | | -. | - | | - | | | - | - | |  |
| 2017 and later | 9 | 90.1 (67.8-99.9) | | 99% | - | | - | | | - | - | |  |
| Sample size |  |  | |  | -2.42 | | .29 | | |  |  | |  |
| <300 | 3 | 98.5 (95.2-99.9) | | 81% | - | | - | | | - | - | |  |
| ≥300 | 6 | 82.7 (55.1-98.5) | | 99% | - | | - | | | - | - | |  |
| Study countries |  |  | |  | - | | - | | | - | - | |  |
| High-income | 0 | - | | - | - | | - | | | - | - | |  |
| Upper-middle income | 9 | 89.0 (68.9-99.3) | | 99% | - | | - | | | - | - | |  |
| Lower-middle income | 0 | - | | - | - | | - | | | - | - | |  |
| Low income | 0 | - | | - | - | | - | | | - | - | |  |
| HIVST along with counseling delivering modes |  |  | |  | - | | - | | | - | - | |  |
| Technology and mobile health | 9 | 89.0 (68.9-99.3) | | 99% | - | | - | | | - | - | |  |
| Peer and community | 0 | - | | - | - | | - | | | - | - | |  |
| HIVST counseling components |  |  | |  | 0.41 | | .86 | | | - | - | |  |
| Post-test counseling | 3 | 87.1 (47.6-99.4) | | 99% | - | | - | | | - | - | |  |
| Both pre-test and post-test counseling | 6 | 93.2 (81.7-99.3) | | 93% | - | | - | | | - | - | |  |
| ***Linkage to laboratory confirmatory that were provided active counseling along with HIVST*** | | | | | | | | | | | | | |
| Year of the study |  |  |  | | | -35.72 | | .98 |  | | |  | |
| 2016 and earlier | 3 | 98.6 (92.7-99.9) | 0% | | | - | | - | - | | | - | |
| 2017 and later | 32 | 87.9 (83.4-91.8) | 71% | | | - | | - | - | | | - | |
| Sample size |  |  |  | | | -1.56 | | **.03** |  | | |  | |
| <300 | 16 | 93.8 (87.5-97.9) | 41% | | | - | | - | - | | | - | |
| ≥300 | 19 | 85.6 (79.6-90.7) | 80% | | | - | | - | - | | | - | |
| Study countries |  |  |  | | | -0.07 | | .32 |  | | |  | |
| High-income | 9 | 87.9 (75.2-96.5) | 0% | | | - | | - | - | | | - | |
| Upper-middle income | 16 | 88.7 (82.0-93.9) | 71% | | | - | | - | - | | | - | |
| Lower-middle income | 6 | 88.9 (76.6-97.0) | 90% | | | - | | - | - | | | - | |
| Low income | 4 | 90.5 (78.4-97.9) | 66% | | | - | | - | - | | | - | |
| HIVST along with counseling delivering modes |  |  |  | | | -0.03 | | .96 |  | | |  | |
| Technology and mobile health | 21 | 88.4 (82.1-93.5) | 60% | | | - | | - | - | | | - | |
| Peer and community | 14 | 88.7 (80.9-94.7) | 81% | | | - | | - | - | | | - | |
| HIVST counseling components |  |  |  | | | 0.54 | | .32 | - | | | - | |
| Post-test counseling | 20 | 88.7 (83.7-92.8) | 70.1% | | | - | | - | - | | | - | |
| Both pre-test and post-test counseling | 15 | 89.4 (79.7-96.2) | 71.1% | | | - | | - | - | | | - | |
| ***Linkage to laboratory confirmatory that were provided passive counseling along with HIVST*** | | | | | | | | | | | | | |
| Year of the study |  |  |  | | | 1.30 | | .27 |  | | |  | |
| 2016 and earlier | 0 | - | -. | | | - | | - | - | | | - | |
| 2017 and later | 5 | 69.3 (31.4-96.1) | 98% | | | - | | - | - | | | - | |
| Sample size |  |  |  | | | 1.30 | | .27 |  | | |  | |
| <300 | 0 | - | - | | | - | | - | - | | | - | |
| ≥300 | 5 | 69.3 (31.4-96.1) | 98% | | | - | | - | - | | | - | |
| Study countries |  |  |  | | | -0.06 | | .96 |  | | |  | |
| High-income | 2 | 59.7 (23.9-90.2) | 91% | | | - | | - | - | | | - | |
| Upper-middle income | 2 | 89.9 (41.6-95.1) | 96% | | | - | | - | - | | | - | |
| Lower-middle income | 1 | - | - | | | - | | - | - | | | - | |
| Low income | 0 | - | - | | | - | | - | - | | | - | |
| HIVST along with counseling delivering modes |  |  |  | | | -1.94 | | .37 |  | | |  | |
| Technology and mobile health | 3 | 99.5 (97.8-99.9) | 0% | | | - | | - | - | | | - | |
| Peer and community | 2 | 51.3 (23.5-78.6) | 88% | | | - | | - | - | | | - | |
| ***Linkage to ART that were provided active counseling along with HIVST*** | | | | | | | | | | | | | |
| Year of the study |  |  |  | | | -16.03 | | .99 |  | | |  | |
| 2016 and earlier | 1 | - | - | | | - | | - | - | | | - | |
| 2017 and later | 20 | 90.0 (87.6-92.1) | 2% | | | - | | - | - | | | - | |
| Sample size |  |  |  | | | 0.15 | | .77 |  | | |  | |
| <300 | 7 | 86.9 (78.8-93.2) | 0% | | | - | | - | - | | | - | |
| ≥300 | 14 | 90.2 (87.2-92.9) | 24% | | | - | | - | - | | | - | |
| Study countries |  |  |  | | | -0.2 | | .45 |  | | |  | |
| High-income | 3 | 93.3 (71.2-99.3) | 0% | | | - | | - | - | | | - | |
| Upper-middle income | 9 | 89.3 (82.4-95.1) | 24% | | | - | | - | - | | | - | |
| Lower-middle income | 6 | 91.2 (88.7-94.3) | 0% | | | - | | - | - | | | - | |
| Low income | 3 | 86.4 (79.3-92.3) | 0% | | | - | | - | - | | | - | |
| HIVST along with counseling delivering modes |  |  |  | | | 0.006 | | .98 |  | | |  | |
| Technology and mobile health | 11 | 89.3 (82.8-94.4) | 18% | | | - | | - | - | | | - | |
| Peer and community | 10 | 87 (79-93) | 79% | | | - | | - | - | | | - | |
| HIVST counseling components |  |  |  | | | 0.0001 | | .99 | - | | | - | |
| Post-test counseling | 9 | 91.4 (89.1-93.4) | 0% | | | - | | - | - | | | - | |
| Both pre-test and post-test counseling | 12 | 85.7 (80.6-90.1) | 0% | | | - | | - | - | | | - | |
| ***Linkage to ART that were provided passive counseling along with HIVST*** | | | | | | | | | | | | | |
| Year of the study |  |  |  | | | 1.32 | | **.05** |  | | |  | |
| 2016 and earlier | 0 | - | -. | | | - | | - | - | | | - | |
| 2017 and later | 4 | 81.6 (67.5-92.4) | 75% | | | - | | - | - | | | - | |
| Sample size |  |  |  | | | - | | **-** |  | | |  | |
| <300 | 0 | - | - | | | - | | - | - | | | - | |
| ≥300 | 4 | 81.6 (67.5-92.4) | 75% | | | - | | - | - | | | - | |
| Study countries |  |  |  | | | 0.26 | | .78 |  | | |  | |
| High-income | 2 | 76.4 (65.3-86.1) | 0% | | | - | | - | - | | | - | |
| Upper-middle income | 2 | 88.3(51.2-99.3) | 91% | | | - | | - | - | | | - | |
| Lower-middle income | 0 | - | - | | | - | | - | - | | | - | |
| Low income | 0 | - | - | | | - | | - | - | | | - | |
| HIVST along with counseling delivering modes |  |  |  | | | 33.8 | | .99 |  | | |  | |
| Technology and mobile health | 3 | 73.7 (66.7-80.2) | 0% | | | - | | - | - | | | - | |
| Peer and community | 1 | - | - | | | - | | - | - | | | - | |
| ***Linkage to physicians that were provided active counseling along with HIVST*** | | | | | | | | | | | | | |
| Year of the study |  |  |  | | | 1.3 | | .20 |  | | |  | |
| 2016 and earlier | 4 | 72.9 (55.4-87.3) | 14% | | | - | | - | - | | | - | |
| 2017 and later | 24 | 91.1 (87.2-94.4) | 28% | | | - | | - | - | | | - | |
| Sample size |  |  |  | | | -0.78 | | .42 |  | | |  | |
| <300 | 15 | 92.0 (83.2-97.7) | 64% | | | - | | - | - | | | - | |
| ≥300 | 13 | 86.0 (82.6-89.0) | 0% | | | - | | - | - | | | - | |
| Study countries |  |  |  | | | -0.10 | | .88 |  | | |  | |
| High-income | 12 | 88.9 (76.5-97.0) | 53% | | | - | | - | - | | | - | |
| Upper-middle income | 14 | 90.4 (84.6-95.0) | 41% | | | - | | - | - | | | - | |
| Lower-middle income | 2 | 92.7 (77.6-99.7) | 61% | | | - | | - | - | | | - | |
| Low income | 0 | - | - | | | - | | - | - | | | - | |
| HIVST along with counseling delivering modes |  |  |  | | | -0.14 | | .89 |  | | |  | |
| Technology and mobile health | 22 | 90.1 (85.9-93.6) | 24% | | | - | | - | - | | | - | |
| Peer and community | 6 | 88.2 (60.5-99.9) | 79% | | | - | | - | - | | | - | |
| HIVST counseling components |  |  |  | | | 1.32 | | .18 | - | | | - | |
| Post-test counseling | 11 | 89.1 (82.1-94.6) | 63% | | | - | | - | - | | | - | |
| Both pre-test and post-test counseling | 17 | 89.5 (83.8-94.1) | 2% | | | - | | - | - | | | - | |
| ***Linkage to physicians that were provided passive counseling along with HIVST*** | | | | | | | | | | | | | |
| Year of the study |  |  |  | | | - | | - | - | | | - | |
| 2016 and earlier | 0 | - | -. | | | - | | - | - | | | - | |
| 2017 and later | 2 | 71.8 (14.3-99.6) | 96% | | | - | | - | - | | | - | |
| Sample size |  |  |  | | | - | | - | - | | | - | |
| <300 | 0 | - | - | | | - | | - | - | | | - | |
| ≥300 | 2 | 71.8 (14.3-99.6) | 96% | | | - | | - | - | | | - | |
| Study countries |  |  |  | | | - | | - | - | | | - | |
| High-income | 2 | 71.8 (14.3-99.6) | 96% | | | - | | - | - | | | - | |
| Upper-middle income | 0 | - | - | | | - | | - | - | | | - | |
| Lower-middle income | 0 | - | - | | | - | | - | - | | | - | |
| Low income | 0 | - | - | | | - | | - | - | | | - | |
| HIVST along with counseling delivering modes |  |  |  | | | - | | - | - | | | - | |
| Technology and mobile health | 2 | 71.8 (14.3-99.6) | 96% | | | - | | - | - | | | - | |
| Peer and community | 0 | - | - | | | - | | - | - | | | - | |
| ***Linkage to MSM with negative HIVST results who received information/intervention related to sexual risk behaviors reduction, pre-exposure prophylaxis (PrEP) or post-exposure prophylaxis (PEP)*** | | | | | | | | | | | | | |
| Year of the study |  |  |  | | | - | | - | - | | | **-** | |
| 2016 and earlier | 1 | - | -. | | | - | | - | - | | | - | |
| 2017 and later | 6 | 98.9 (91.7-99.4) | 99% | | | - | | - | - | | | - | |
| Sample size |  |  |  | | | -810.62 | | **.04** | -1257.77 | | | **0.0003** | |
| <300 | 3 | 99.8 (99.0-99.9) | 0% | | | - | | - | - | | | - | |
| ≥300 | 4 | 98.4 (87.4-98.8) | 99% | | | - | | - | - | | | - | |
| Study countries |  |  |  | | | 39.82 | | .93 |  | | |  | |
| High-income | 2 | 94.2 (66.4-98.2) | 98% | | | - | | - | - | | | - | |
| Upper-middle income | 5 | 99.9 (99.7-100.0) | 0% | | | - | | - | - | | | - | |
| Lower-middle income | 0 | - | - | | | - | | - | - | | | - | |
| Low income | 0 | - | - | | | - | | - | - | | | - | |
| HIVST along with counseling delivering modes |  |  |  | | | 572.44 | | **.002** | 51.25 | | | 0.72 | |
| Technology and mobile health | 5 | 98.8 (90.6-99.2) | 99% | | | - | | - | - | | | - | |
| Peer and community | 2 | 99.7 (98.2-99.9) | 0% | | | - | | - | - | | | - | |
| HIVST counseling components |  |  |  | | | - | | - | - | | | - | |
| Post-test counseling | 0 | - | - | | | - | | - | - | | | - | |
| Both pre-test and post-test counseling | 7 | 99.9 (99.8-99.9) | 0% | | | - | | - | - | | | - | |
| ***Linkage to starting PrEP that were provided active counseling along with HIVST*** | | | | | | | | | | | | | |
| Year of the study |  |  |  | | | -0.99 | | **.08** |  | | |  | |
| 2016 and earlier | 0 | - | -. | | | - | | - | - | | | - | |
| 2017 and later | 6 | 27.0 (10.2-54.6) | 97% | | | - | | - | - | | | - | |
| Sample size |  |  |  | | | -0.56 | | .56 | - | | | - | |
| <300 | 3 | 35.6 (5.4-74.6) | 96% | | | - | | - | - | | | - | |
| ≥300 | 3 | 23.9 (8.5-44.0) | 75% | | | - | | - | - | | | - | |
| Study countries |  |  |  | | | -0.43 | | .49 | - | | | - | |
| High-income | 4 | 37.3 (7.5-74.2) | 99% | | | - | | - | - | | | - | |
| Upper-middle income | 1 | - | - | | | - | | - | - | | | - | |
| Lower-middle income | 1 | - | - | | | - | | - | - | | | - | |
| Low income | 0 | - | - | | | - | | - | - | | | - | |
| HIVST along with counseling delivering modes |  |  |  | | | 0.14 | | .88 | - | | | - | |
| Technology and mobile health | 3 | 28.6 (4.3-63.3) | 97% | | | - | | - | - | | | - | |
| Peer and community | 3 | 30.7 (7.4-61.3) | 98% | | | - | | - | - | | | - | |
| HIVST counseling components |  |  |  | | | 1.28 | | .18 | - | | | - | |
| Post-test counseling | 4 | 20.2 (8.0-36.2) | 97% | | | - | | - | - | | | - | |
| Both pre-test and post-test counseling | 2 | 53.0 (29.3-76.0) | 79% | | | - | | - | - | | | - | |

^1^ Pooled estimate not performed due to less than two studies; multivariable meta-regression not performed due to less than two significant (*p*<.10) factors in the univariate analysis
